# Supplementary material for: Caliciviral protein-based artificial translational activator for mammalian gene circuits with RNA-only delivery
Source: Nat Commun. 2020 Mar 10;11:1297. doi: 10.1038/s41467-020-15061-x (PMC7064597; doi:10.1038/s41467-020-15061-x)
Supplement: Supplementary file 1 — Supplementary Information [file 41467_2020_15061_MOESM1_ESM.pdf]

Supplementary Information

Caliciviral protein-based artificial translational activator for mammalian gene circuits with RNA-only delivery

Hideyuki Nakanishi and Hirohide Saito

Contents

Supplementary Figures

1 | Effects of MS2CP-unfused VPg on the translation of synthetic mRNAs.

2 | Effect of CaVT on the translation of an ARCA-capped synthetic mRNA.

3 | Representative two-dimensional dot plots showing the effects of CaVT on the translation of target mRNA variants (target mRNA : CaVT = 9 : 1).

4 | Effects of CaVT on the translation of target mRNA variants (target mRNA : CaVT = 4 : 1).

5 | Representative two-dimensional dot plots showing the effects of CaVT on the translation of target mRNA variants (target mRNA : CaVT = 4 : 1).

6 | Representative two-dimensional dot plots showing the effects of CaVT on the translation of 2xScMS2(C)-hmAG1 mRNA (A-capped target mRNA : CaVT = 9 : 1, ARCA-capped target mRNA : CaVT = 1 : 2).

7 | Effects of CaVT on the translation of 2xScMS2(C)-hmAG1 mRNA (A-capped target mRNA : CaVT = 4 : 1).

8 | Viability of HeLa cells transfected with miRNA-responsive, apoptosis-inducing gene circuits.

9 | Representative dot plots of HeLa cells transfected with miRNA-responsive, apoptosis-inducing gene circuits.

10 | Representative histograms of EGFP knockout by CRISPR/Cas9 system regulated by miRNA-responsive CaVT.

11 | Representative histograms showing the effects of drug-regulatable CaVT on the translation of target mRNA variants.

12 | Representative two-dimensional dot plots showing the effects of drug-regulatable CaVT on the translation of target mRNA variants.

13 | Representative two-dimensional dot plots showing MS2CP-1xDmrA and DmrC-VPg(FCV) dependency of A/C heterodimerizer-mediated translational activation.

14 | Representative two-dimensional dot plots showing the dose-dependency of drug-regulatable CaVT.

Supplementary Table

1 | Exact P values of Figure 5b-c; 6b; 7b, 7d

Supplementary Methods

・ Detailed procedures of the pDNA construction

・ Full sequences of template DNAs for in vitro transcription

・ List of primers to prepare templates for in vitro transcription

・ List of transfection conditions

・Gating strategy

Supplementary Figures

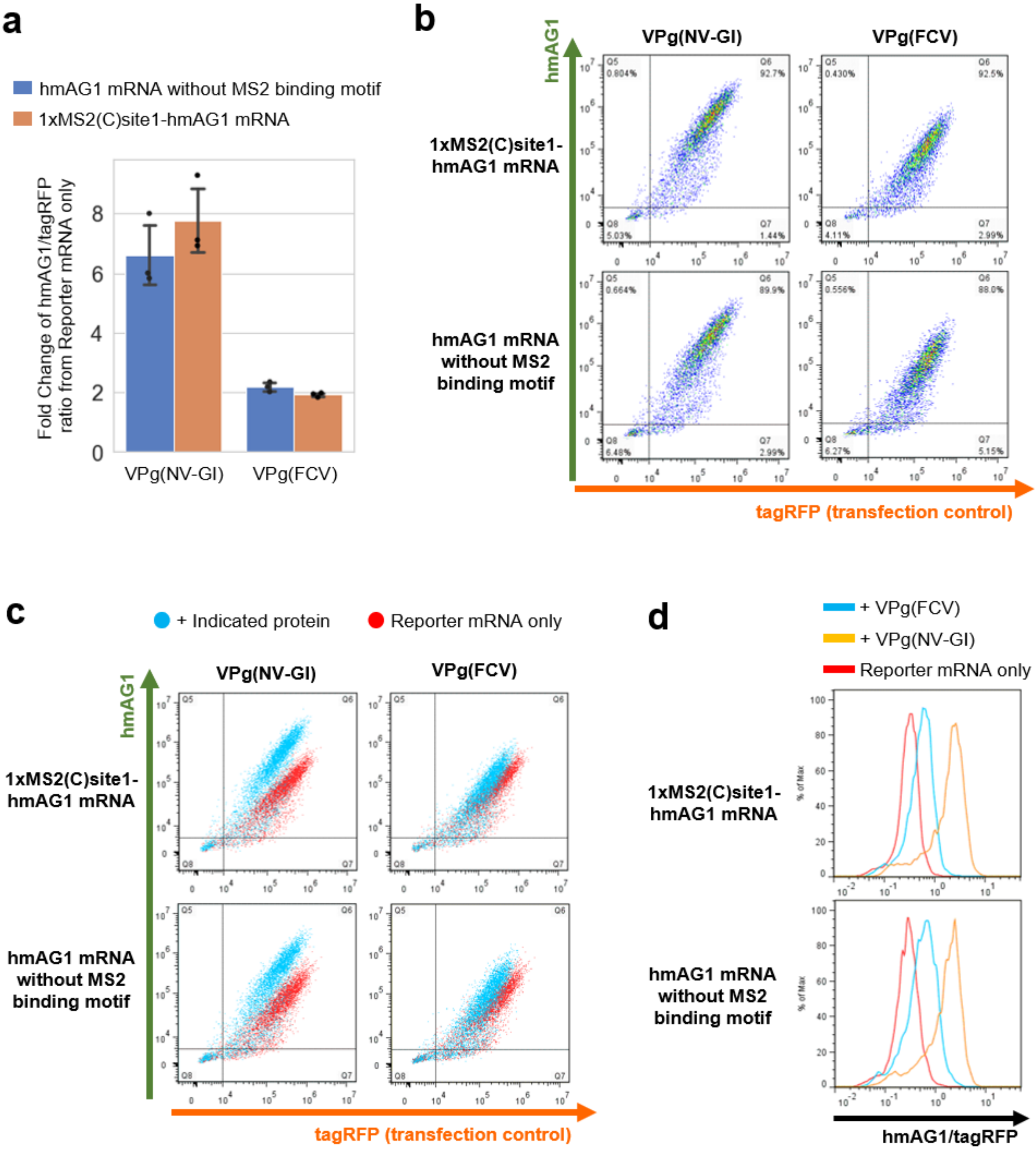

Supplementary Figure 1 | Effects of MS2CP-unfused VPg on the translation of synthetic mRNAs.
HeLa cells were co-transfected with target hmAG1 mRNA and the mRNA of each indicated protein. Fluorescence was measured by a flow cytometer. (a) Fold change of the hmAG1/tagRFP ratio caused by each indicated protein. Cells expressing both hmAG1 and tagRFP were used to calculate the hmAG1/tagRFP ratio, and the average of three independent experiments are shown. The bar graph shows mean ± SD. Source data are provided as a Source Data file. (b) Representative two-dimensional dot plots of hmAG1 and tagRFP. (c) Superimposition of the dot plots shown in (b). Cells transfected with mRNA to express the indicated proteins are shown as cyan, while cells transfected with only reporter mRNAs are shown as red. (d) Representative histograms of the hmAG1/tagRFP ratio in cells expressing both hmAG1 and tagRFP. The details of the transfection conditions are shown in the supplementary methods.

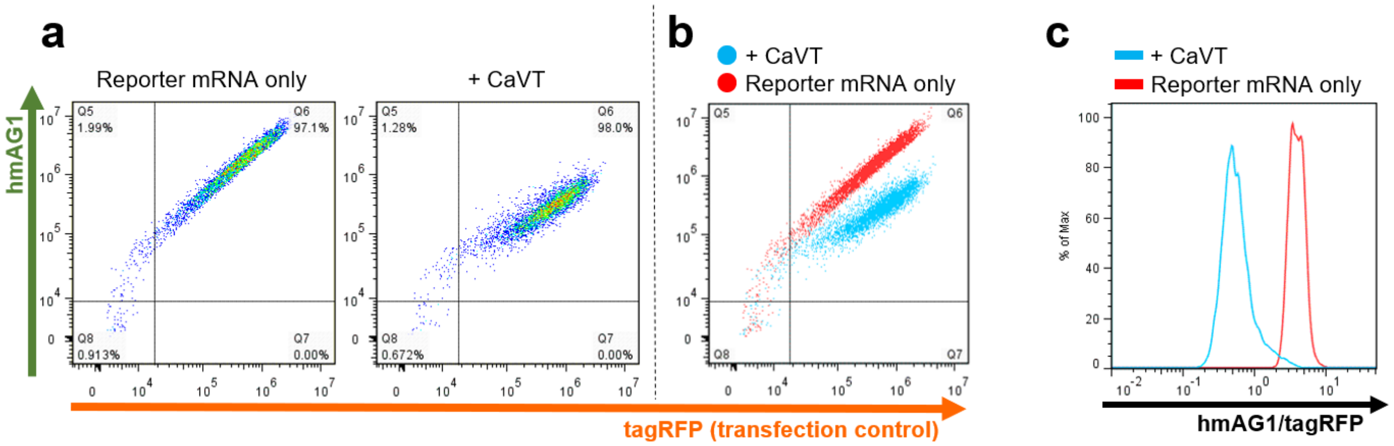

Supplementary Figure 2 | Effect of CaVT on the translation of an ARCA-capped synthetic mRNA.
HeLa cells were co-transfected with 1xMS2(C)site1-hmAG1 mRNA (cap analog: ARCA), tagRFP mRNA, and CaVT mRNA. Fluorescence was measured by a flow cytometer. (a) Representative two-dimensional dot plots of hmAG1 and tagRFP. (b) Superimposition of the dot plots shown in (a). Cells transfected with mRNA to express the indicated proteins are shown as cyan, and cells transfected with only reporter mRNAs are shown as red. (c) Representative histograms of the hmAG1/tagRFP ratio in cells expressing both hmAG1 and tagRFP. The details of the transfection conditions are shown in the supplementary methods.

a

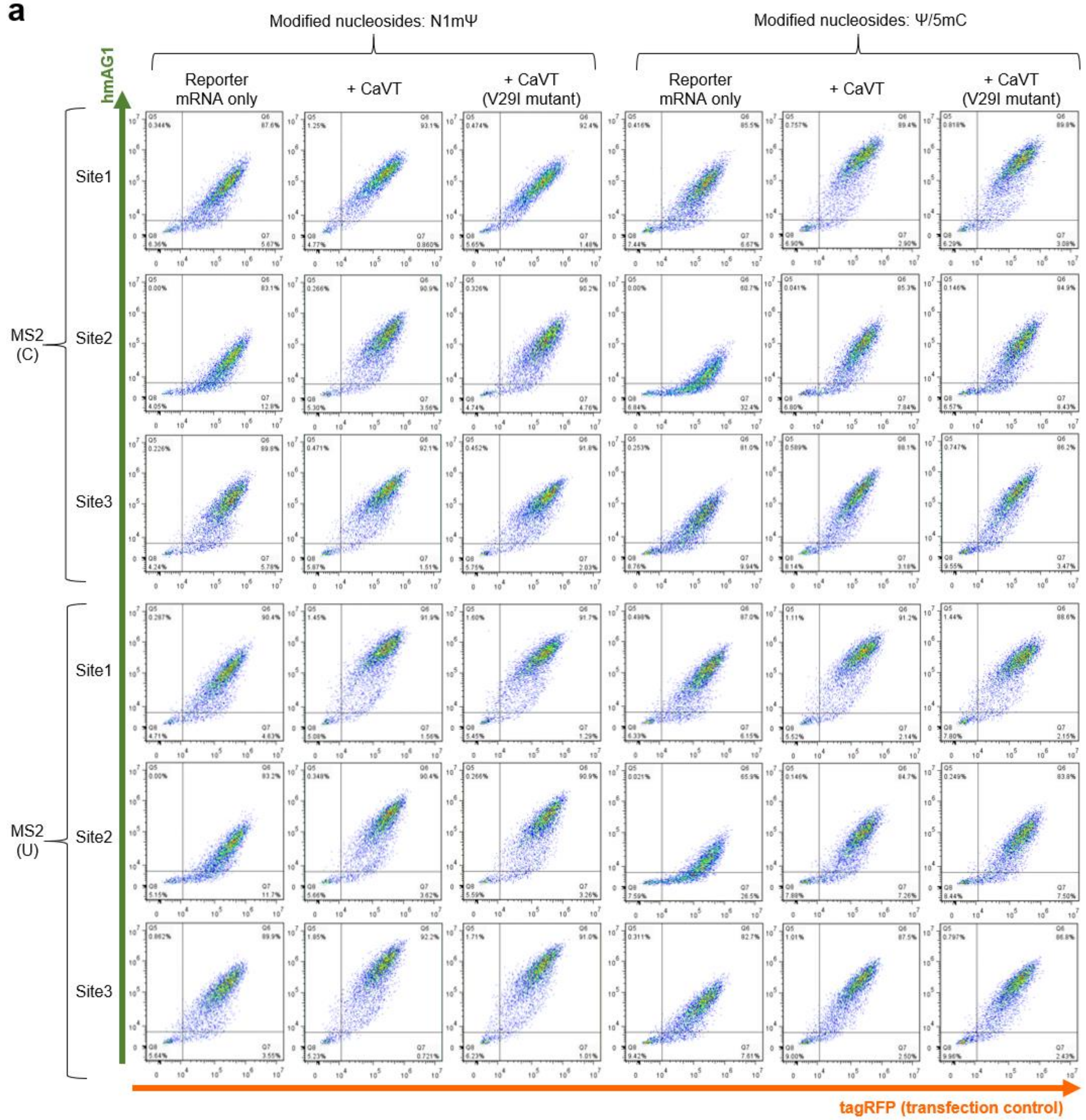

Modified nucleosides: N1mΨ
Modified nucleosides: Ψ/5mC
Reporter mRNA only
+ CaVT
+ CaVT (V29I mutant)
hmAG1
MS2 (C)
MS2 (U)
Site1
Site2
Site3
tagRFP (transfection control)

b

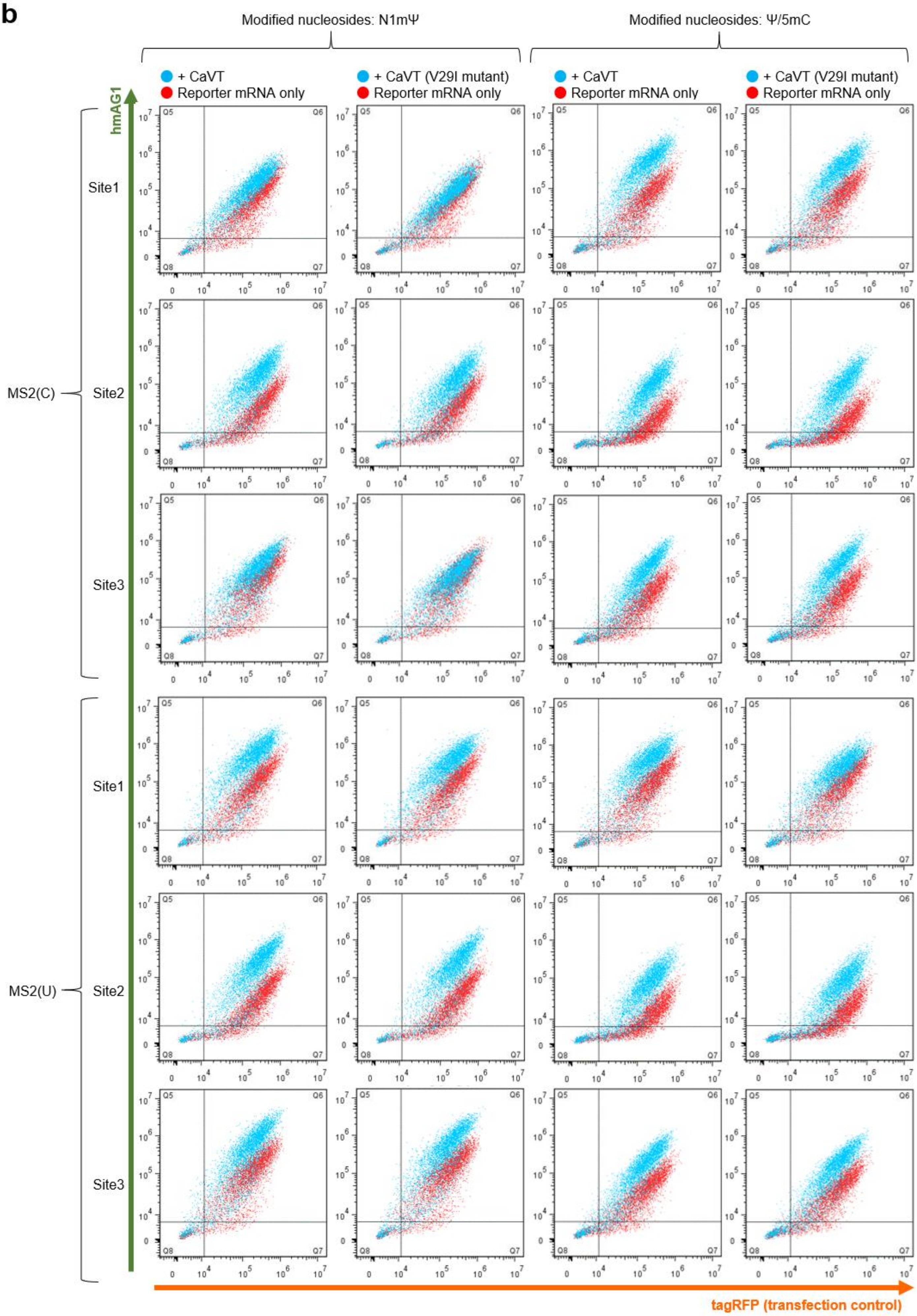
Modified nucleosides: N1mΨ
Modified nucleosides: Ψ/5mC
+ CaVT
+ CaVT (V29I mutant)
Reporter mRNA only
hmAG1
tagRFP (transfection control)
MS2(C)
MS2(U)
Site1
Site2
Site3
Q5
Q6
Q7
Q8

Supplementary Figure 3 | Representative two-dimensional dot plots showing the effects of CaVT on the translation of target mRNA variants (target mRNA : CaVT = 9 : 1).

HeLa cells were co-transfected with each target hmAG1 mRNA variant and CaVT (or its V29I mutant) mRNA. Fluorescence was measured by a flow cytometer. The superimpositions of (a) are shown in (b). The histograms of the hmAG1 (+)/tagRFP (+) population are shown in Figure 3c. Details of the transfection conditions are shown in the supplementary methods.

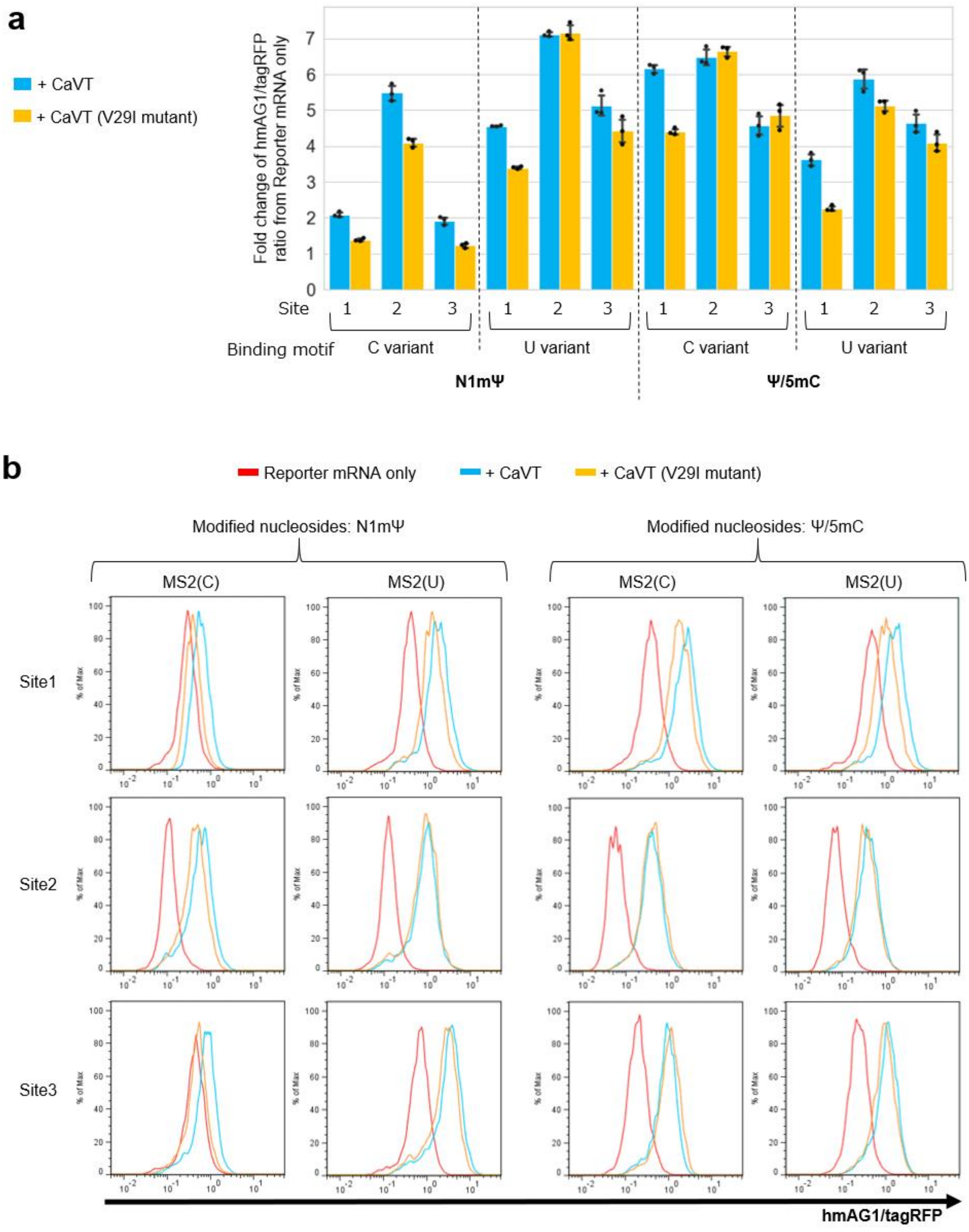

Supplementary Figure 4 | Effects of CaVT on the translation of target mRNA variants (target mRNA : CaVT = 4 : 1).

HeLa cells were co-transfected with hmAG1 mRNAs containing MS2 binding motifs (cap analog: A-cap), tagRFP mRNA (cap analog: ARCA), and mRNA that expresses CaVT or its V29I mutant (cap analog: ARCA). One day

after the transfection, the fluorescence of hmAG1 and tagRFP was measured by a flow cytometer. (a) CaVT (or its V29I mutant)-mediated fold change of the hmAG1/tagRFP ratio in cells transfected with the indicated reporter mRNAs. Cells expressing both hmAG1 and tagRFP were used to calculate the hmAG1/tagRFP ratio, and the average of three independent experiments is shown. The bar graph shows mean ± SD. Source data are provided as a Source Data file. (b) Representative histograms of the hmAG1/tagRFP ratio in cells expressing both hmAG1 and tagRFP. Cells transfected with only reporter mRNAs are shown as red, and cells transfected with mRNA that expresses CaVT or its V29I mutant are shown as cyan and orange, respectively. Details of the transfection conditions are shown in the supplementary methods.

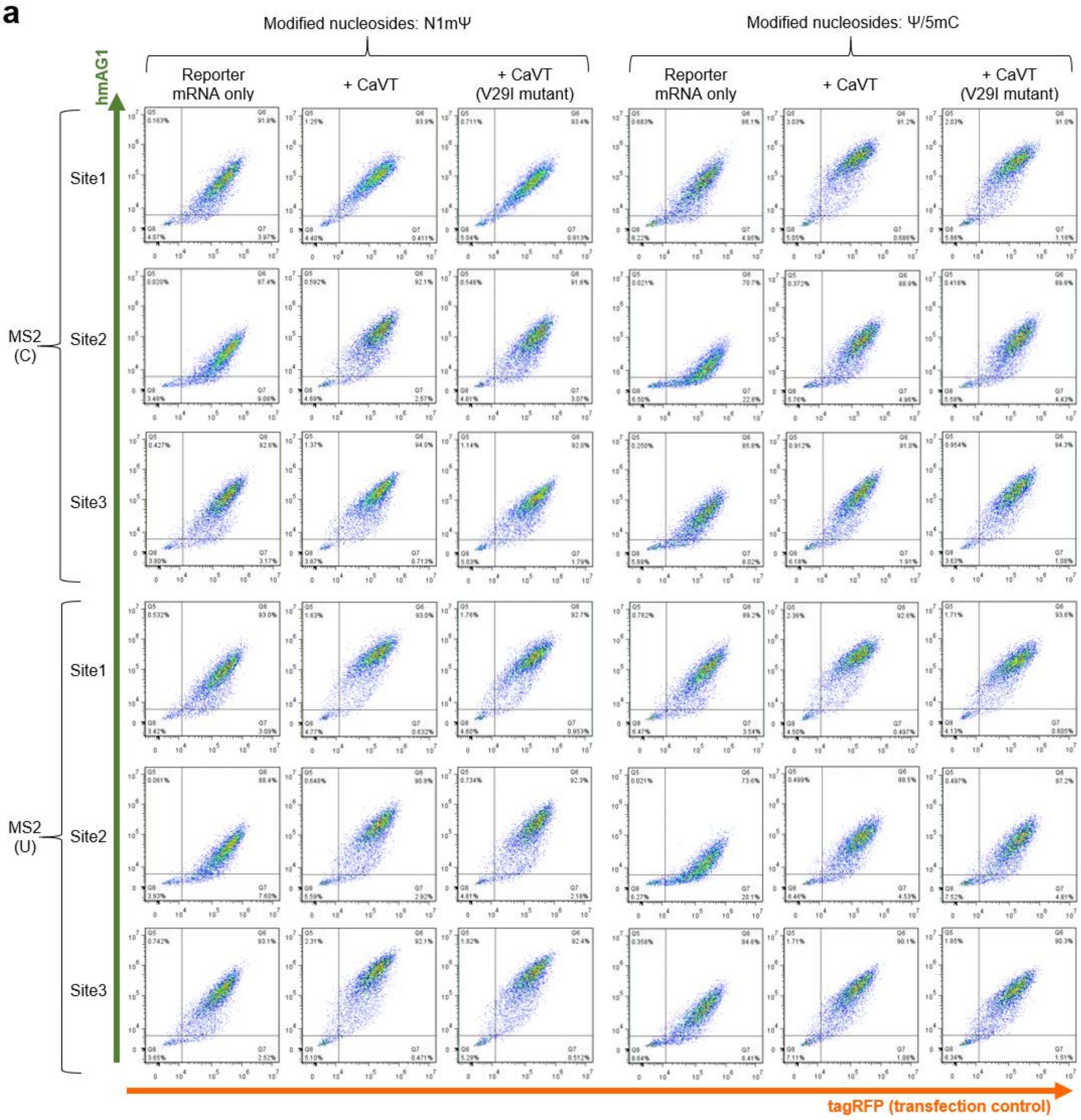

b

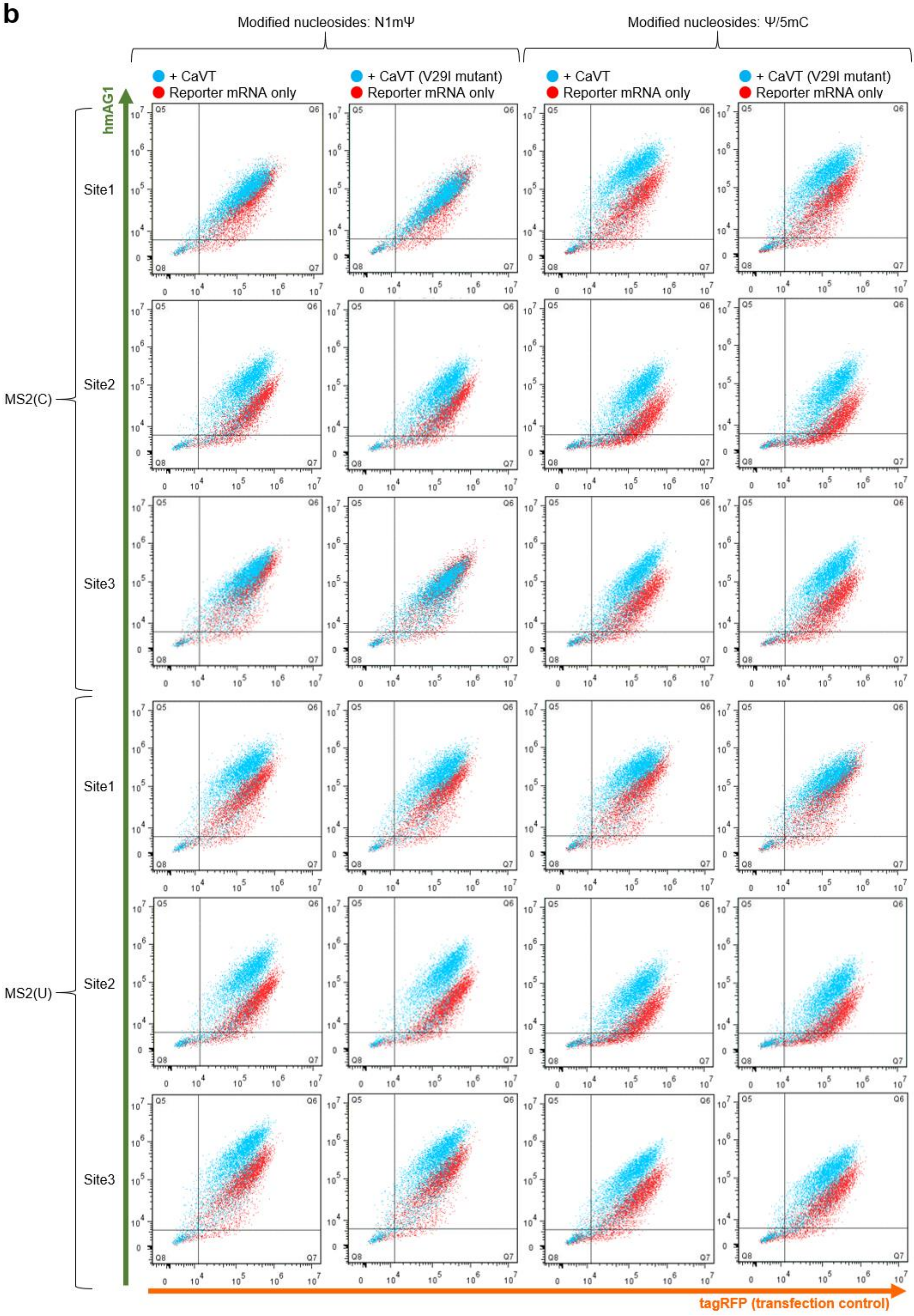
Modified nucleosides: N1mΨ
Modified nucleosides: Ψ/5mC
+ CaVT
Reporter mRNA only
+ CaVT (V29I mutant)
Reporter mRNA only
hmAG1
MS2(C)
MS2(U)
Site1
Site2
Site3
Q5
Q6
Q7
Q8
tagRFP (transfection control)

Supplementary Figure 5 | Representative two-dimensional dot plots showing the effects of CaVT on the translation of target mRNA variants (target mRNA : CaVT = 4 : 1).

HeLa cells were co-transfected with each target hmAG1 mRNA variant and CaVT (or its V29I mutant) mRNA. Fluorescence was measured by a flow cytometer. The superimpositions of (a) are shown in (b). Details of the experimental procedure are described in the supplementary methods. The histograms of the hmAG1 (+)/tagRFP (+) population are shown in Supplementary Figure 4b.

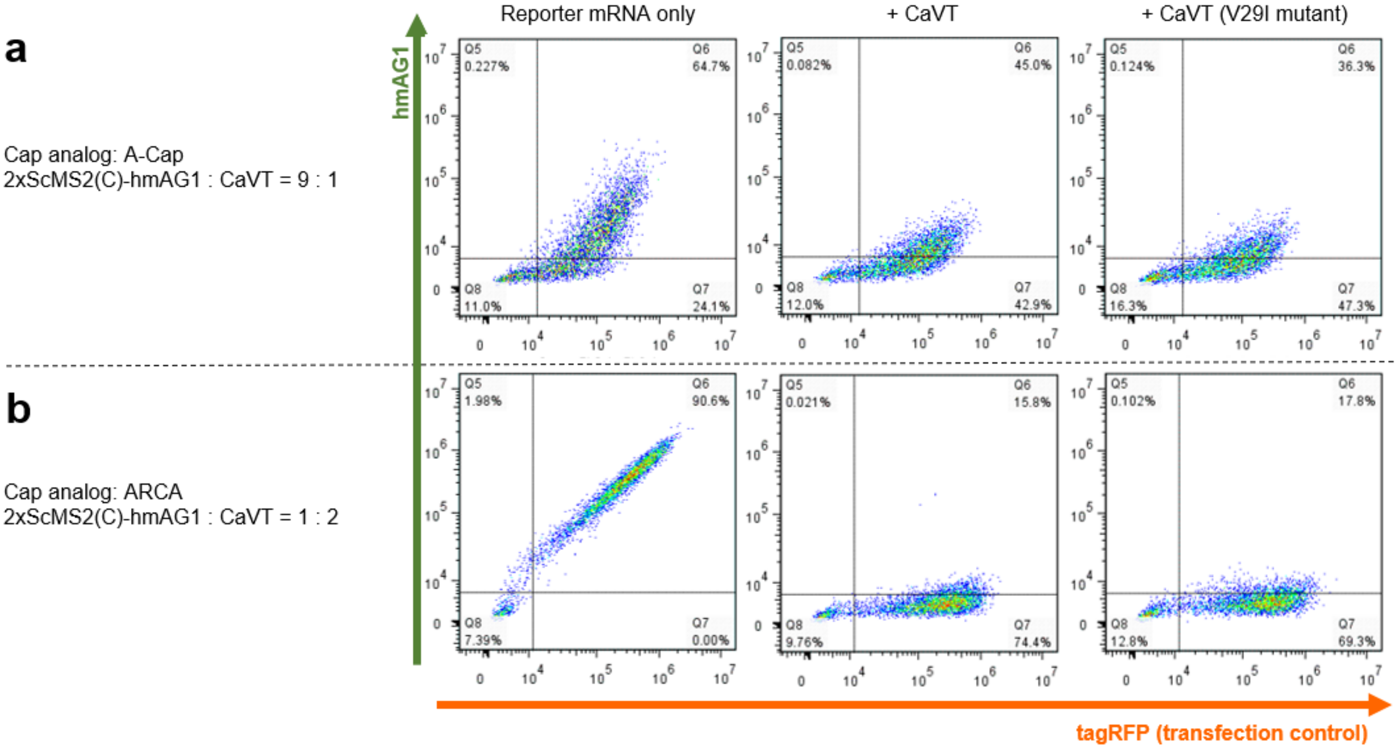

Supplementary Figure 6 | Representative two-dimensional dot plots showing the effects of CaVT on the translation of 2xScMS2(C)-hmAG1 mRNA (A-capped target mRNA : CaVT = 9 : 1, ARCA-capped target mRNA : CaVT = 1 : 2).

HeLa cells were co-transfected with A-capped (top) or ARCA-capped (bottom) 2xScMS2(C)-hmAG1 mRNAs, tagRFP mRNA, and mRNA that expresses CaVT or its V29I mutant. One day after the transfection, the fluorescence of hmAG1 and tagRFP was measured by a flow cytometer. Details of the transfection conditions are shown in the supplementary methods.

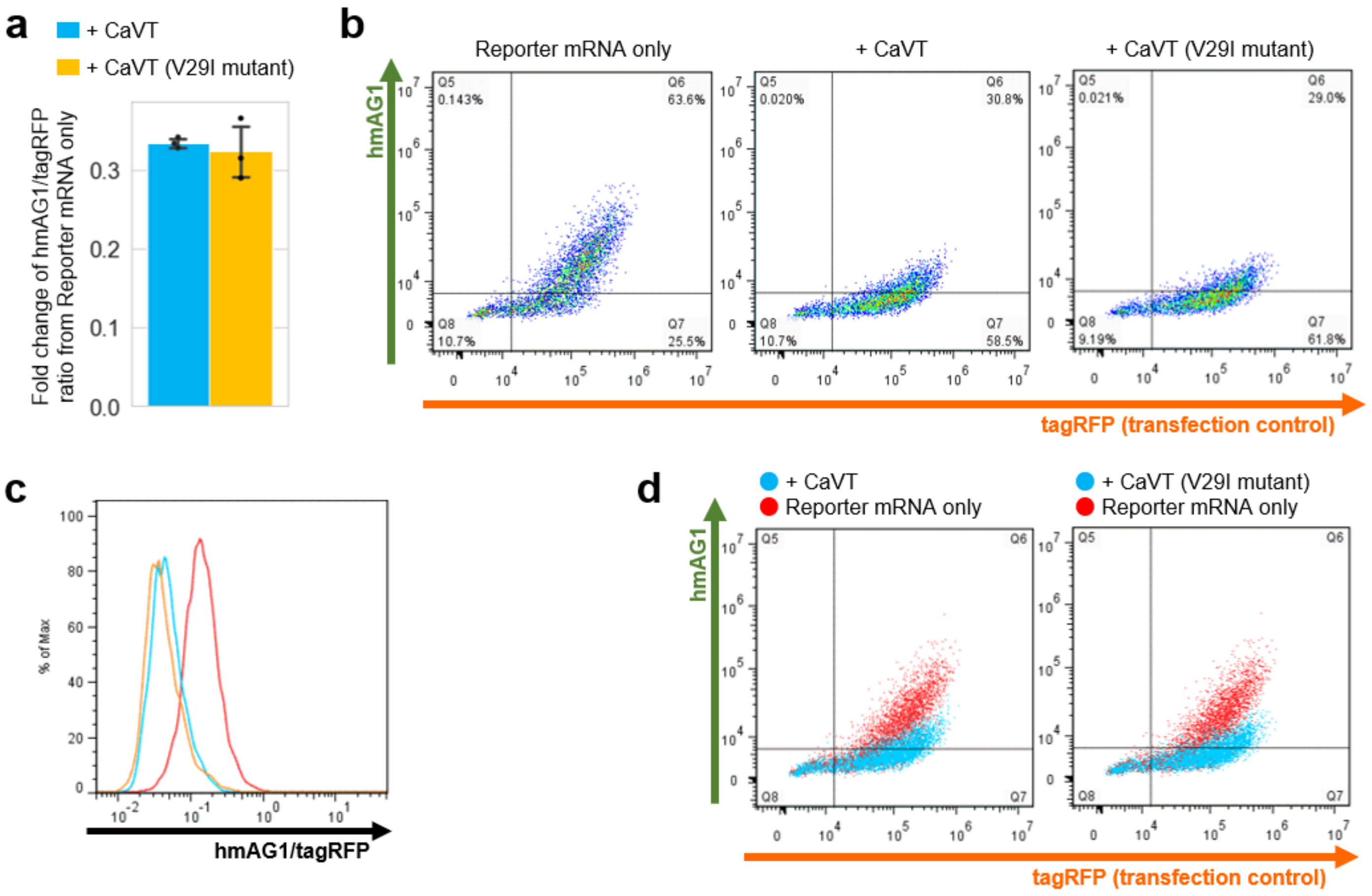

Supplementary Figure 7 | Effects of CaVT on the translation of 2xScMS2(C)-hmAG1 mRNA (A-capped target mRNA : CaVT = 4 : 1).

HeLa cells were co-transfected with A-capped 2xScMS2(C)-hmAG1 mRNAs, tagRFP mRNA, and mRNA that expresses CaVT or its V29I mutant. One day after the transfection, the fluorescence of hmAG1 and tagRFP was measured by a flow cytometer. (a) Fold change of the hmAG1/tagRFP ratio caused by each indicated protein. Means of the hmAG1/tagRFP ratio were normalized by the hmAG1/tagRFP ratio in the reporter mRNA only sample. The bar graph shows the average of three independent experiments (mean ± SD). Source data are provided as a Source Data file. (b) Representative two-dimensional dot plots of hmAG1 and tagRFP. (c) Representative histograms of the hmAG1/tagRFP ratio in cells expressing both hmAG1 and tagRFP. (d) Superimposition of the dot plots shown in (b). Cells transfected with mRNA to express the indicated proteins are shown as cyan, and cells transfected with only reporter mRNAs are shown as red. Details of the transfection conditions are shown in the supplementary methods.

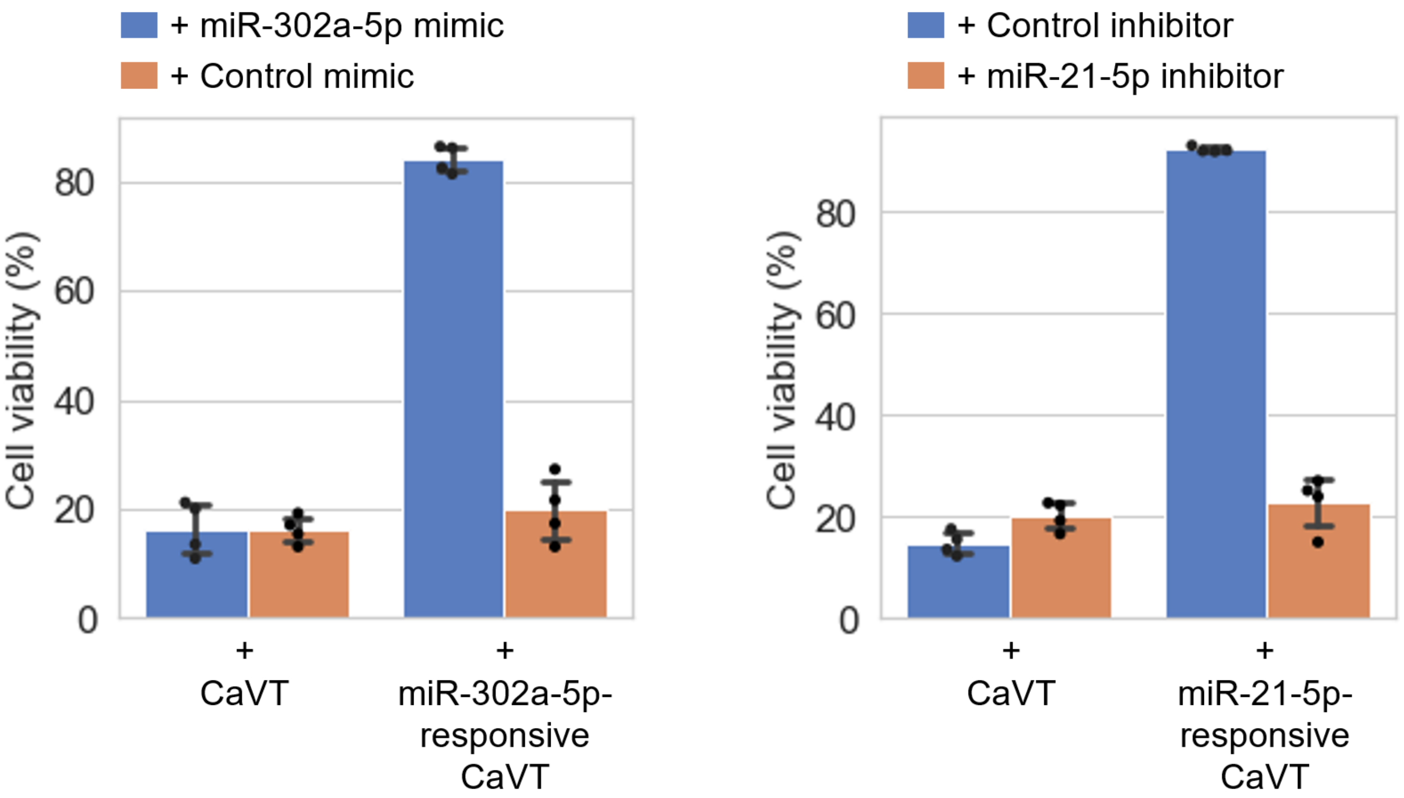

Supplementary Figure 8 | Viability of HeLa cells transfected with miRNA-responsive, apoptosis-inducing gene circuits.

HeLa cells were co-transfected with 1xMS2(U)site2-Bax mRNA (cap analog: A-cap, modified nucleosides: N1mΨ), 2xScMS2(C)-BclxL mRNA (cap analog: ARCA, modified nucleosides: N1mΨ), the indicated CaVT mRNA, and the indicated miRNA mimic or inhibitor. One day after the transfection, cell viability was measured by the WST-1 assay. The bar graphs show mean ± SD (n = 4 independent wells in each transfection condition). Details of the transfection conditions are shown in the supplementary methods. Source data are provided as a Source Data file.

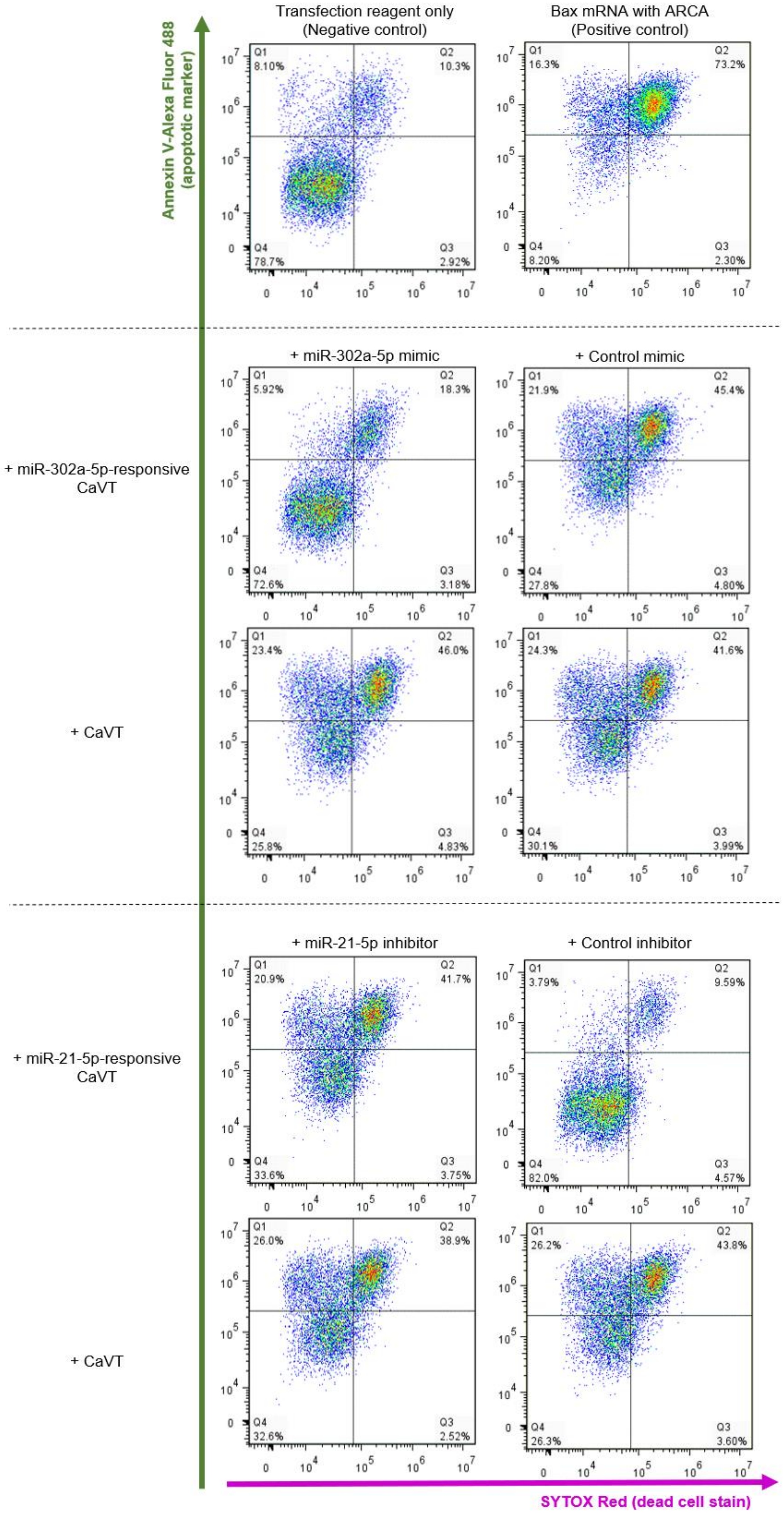
Annexin V-Alexa Fluor 488 (apoptotic marker)
SYTOX Red (dead cell stain)
Transfection reagent only (Negative control)
Q1 8.10%
Q2 10.3%
Q4 78.7%
Q3 2.92%
Bax mRNA with ARCA (Positive control)
Q1 16.3%
Q2 73.2%
Q4 8.20%
Q3 2.30%
+ miR-302a-5p-responsive CaVT
+ miR-302a-5p mimic
Q1 5.92%
Q2 18.3%
Q4 72.6%
Q3 3.18%
+ Control mimic
Q1 21.9%
Q2 45.4%
Q4 27.8%
Q3 4.80%
+ CaVT
Q1 23.4%
Q2 46.0%
Q4 25.8%
Q3 4.83%
Q1 24.3%
Q2 41.6%
Q4 30.1%
Q3 3.99%
+ miR-21-5p-responsive CaVT
+ miR-21-5p inhibitor
Q1 20.9%
Q2 41.7%
Q4 33.6%
Q3 3.75%
+ Control inhibitor
Q1 3.79%
Q2 9.59%
Q4 82.0%
Q3 4.57%
+ CaVT
Q1 26.0%
Q2 38.9%
Q4 32.6%
Q3 2.52%
Q1 26.2%
Q2 43.8%
Q4 26.3%
Q3 3.60%

Supplementary Figure 9 | Representative dot plots of HeLa cells transfected with miRNA-responsive, apoptosis-inducing gene circuits.

HeLa cells were co-transfected with 1xMS2(U)site2-Bax mRNA (cap analog: A-cap, modified nucleosides: N1mΨ), 2xScMS2(C)-BclxL mRNA (cap analog: ARCA, modified nucleosides: N1mΨ), the indicated CaVT mRNA, and the indicated miRNA mimic or inhibitor. One day after the transfection, the cells were stained by Annexin V and SYTOX Red, followed by the measurement of fluorescence by a flow cytometer. Details of the transfection conditions are shown in the supplementary methods.

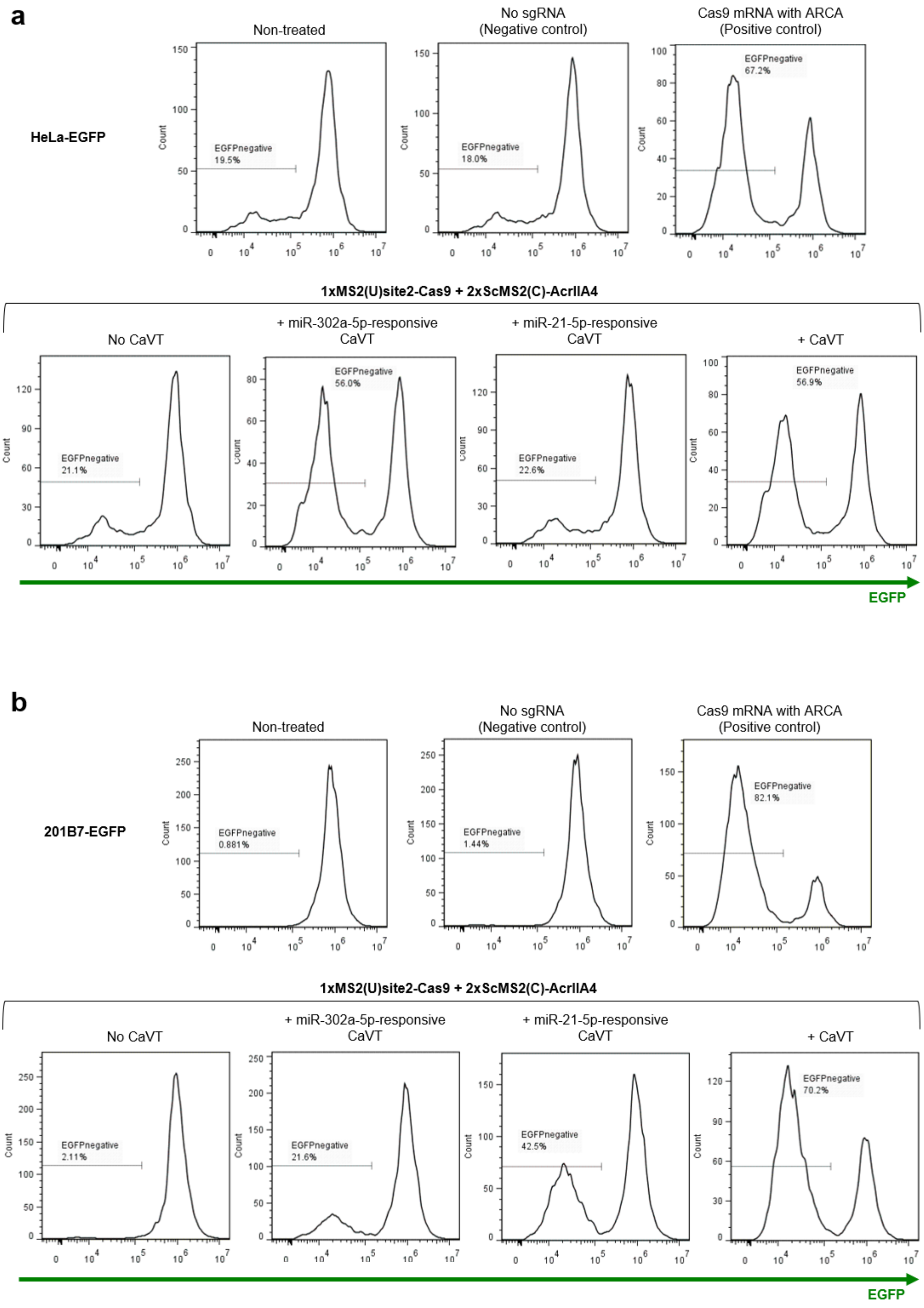
a
Non-treated
No sgRNA
(Negative control)
Cas9 mRNA with ARCA
(Positive control)
HeLa-EGFP
EGFPnegative
19.5%
EGFPnegative
18.0%
EGFPnegative
67.2%
1xMS2(U)site2-Cas9 + 2xScMS2(C)-AcrIIA4
No CaVT
+ miR-302a-5p-responsive
CaVT
+ miR-21-5p-responsive
CaVT
+ CaVT
EGFPnegative
21.1%
EGFPnegative
56.0%
EGFPnegative
22.6%
EGFPnegative
56.9%
Count
EGFP
b
Non-treated
No sgRNA
(Negative control)
Cas9 mRNA with ARCA
(Positive control)
201B7-EGFP
EGFPnegative
0.881%
EGFPnegative
1.44%
EGFPnegative
82.1%
1xMS2(U)site2-Cas9 + 2xScMS2(C)-AcrIIA4
No CaVT
+ miR-302a-5p-responsive
CaVT
+ miR-21-5p-responsive
CaVT
+ CaVT
EGFPnegative
2.11%
EGFPnegative
21.6%
EGFPnegative
42.5%
EGFPnegative
70.2%
Count
EGFP

Supplementary Figure 10 | Representative histograms of EGFP knockout by CRISPR/Cas9 system regulated by miRNA-responsive CaVT.

HeLa cells (a) and iPS cells (201B7 strain) (b) were co-transfected with 1xMS2(U)site2-Cas9 mRNA (cap analog: A-cap, modified nucleosides: N1mΨ), 2xScMS2(C)-AcrIIA4 mRNA (cap analog: ARCA, modified nucleosides: N1mΨ), EGFP-targeting sgRNA, and the indicated CaVT mRNA. Five days after the transfection, the fluorescence was measured by a flow cytometer. Details of the transfection conditions are shown in the supplementary methods.

	Drug-regulatable CaVT	A/C heterodimerizer (nM)
	+ (V29I mutant)	500
	+ (V29I mutant)	0
	+ (Normal)	500
	+ (Normal)	0
	−	0

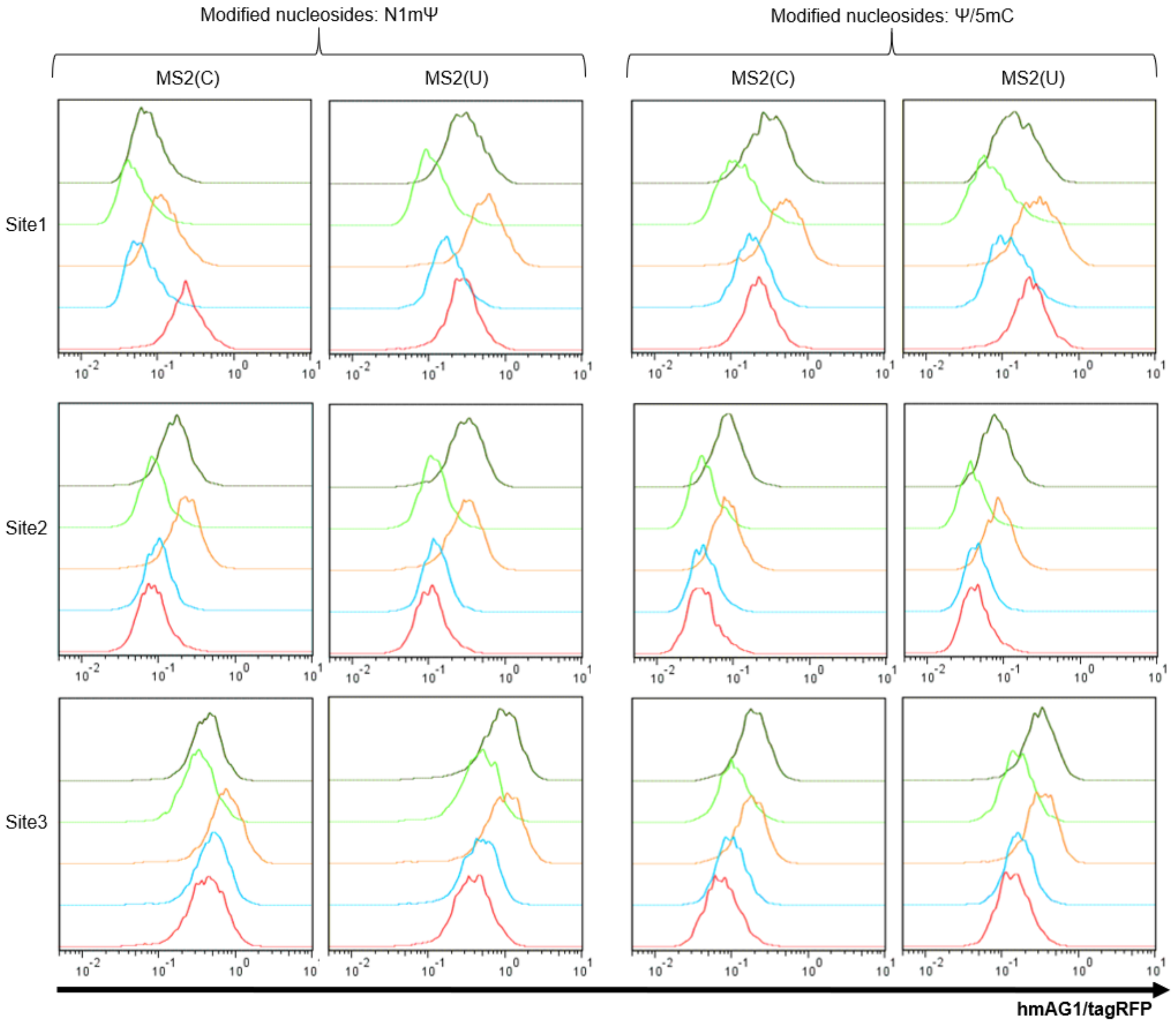

Supplementary Figure 11 | Representative histograms showing effects of drug-regulatable CaVT on the translation of target mRNA variants.

HeLa cells were co-transfected with each target hmAG1 mRNA variant, DmrC-VPg(FCV) mRNA, and MS2CP-1xDmrA (or MS2CP(V29I)-1xDmrA) mRNA. After the transfection, the cells were cultured in medium containing 0 or 500 nM A/C heterodimerizer, and the fluorescence was measured by a flow cytometer. Histograms show the hmAG1 (+)/tagRFP (+) populations. Details of the transfection conditions are shown in the supplementary methods.

a

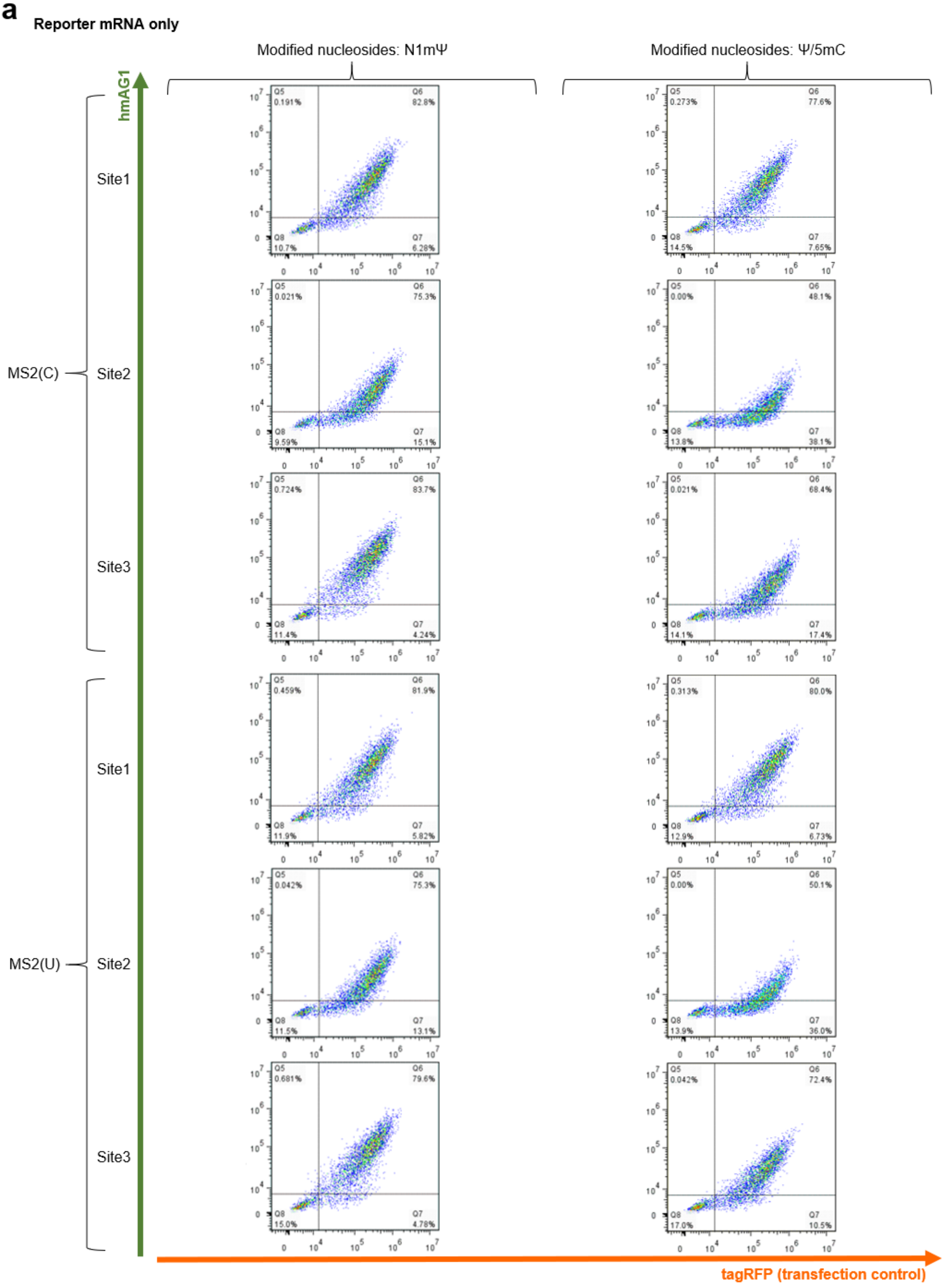

Reporter mRNA only
Modified nucleosides: N1mΨ
Modified nucleosides: Ψ/5mC
hmAG1
MS2(C)
MS2(U)
Site1
Site2
Site3
Q5
Q6
Q7
Q8
0.191%
82.8%
10.7%
6.28%
0.273%
77.6%
14.5%
7.65%
0.021%
75.3%
9.59%
15.1%
0.00%
48.1%
13.8%
38.1%
0.724%
83.7%
11.4%
4.24%
0.021%
68.4%
14.1%
17.4%
0.459%
81.9%
11.9%
5.82%
0.313%
80.0%
12.9%
6.73%
0.042%
75.3%
11.5%
13.1%
0.00%
50.1%
13.9%
36.0%
0.681%
79.6%
15.0%
4.78%
0.042%
72.4%
17.0%
10.5%
tagRFP (transfection control)

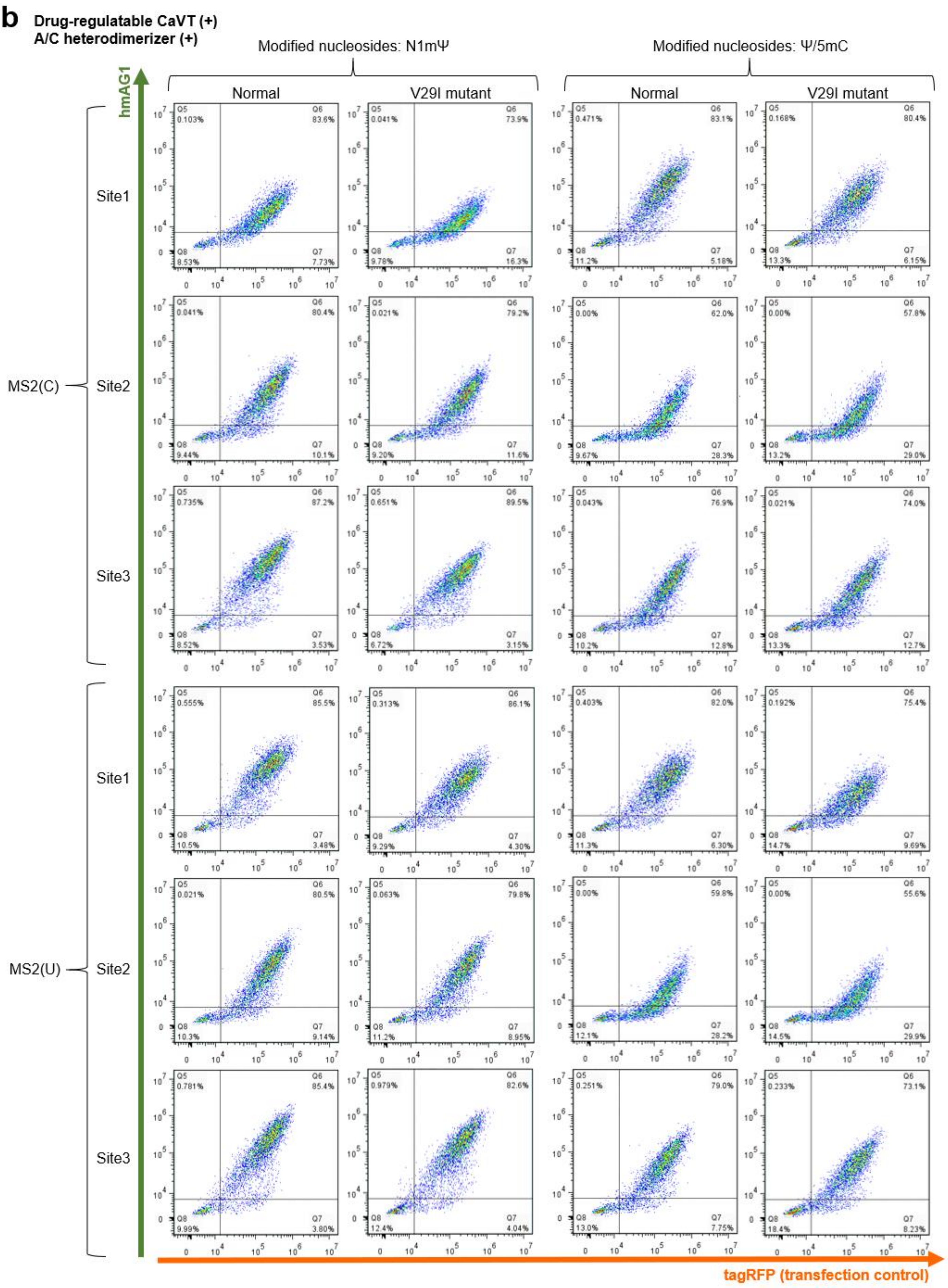
b
Drug-regulatable CaVT (+)
A/C heterodimerizer (+)
Modified nucleosides: N1mΨ
Modified nucleosides: Ψ/5mC
Normal
V29I mutant
Normal
V29I mutant
hmAG1
MS2(C)
MS2(U)
Site1
Site2
Site3
Site1
Site2
Site3
tagRFP (transfection control)

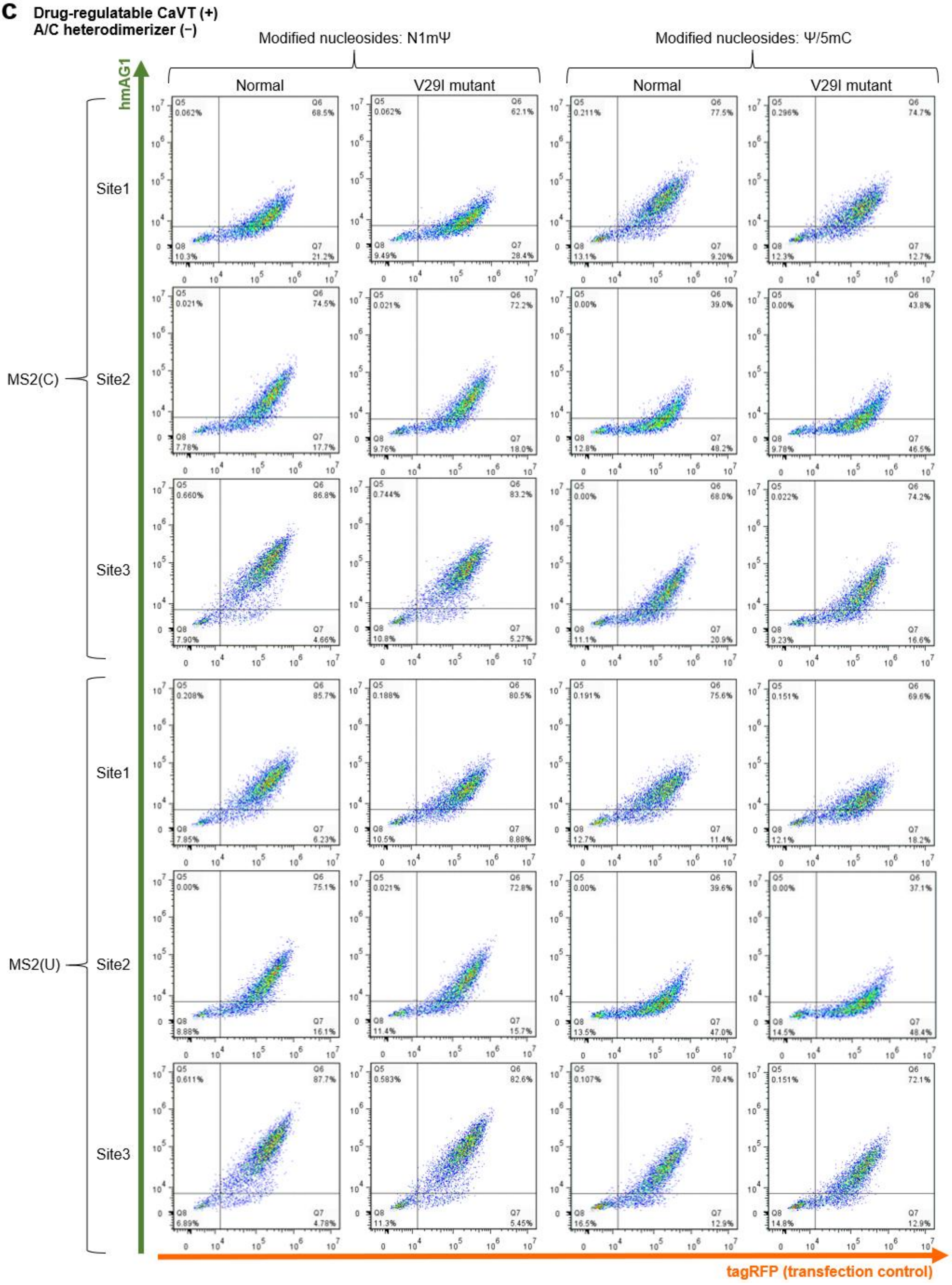
c
Drug-regulatable CaVT (+)
A/C heterodimerizer (−)
Modified nucleosides: N1mΨ
Modified nucleosides: Ψ/5mC
Normal
V29I mutant
Normal
V29I mutant
hmAG1
MS2(C)
MS2(U)
Site1
Site2
Site3
Site1
Site2
Site3
tagRFP (transfection control)

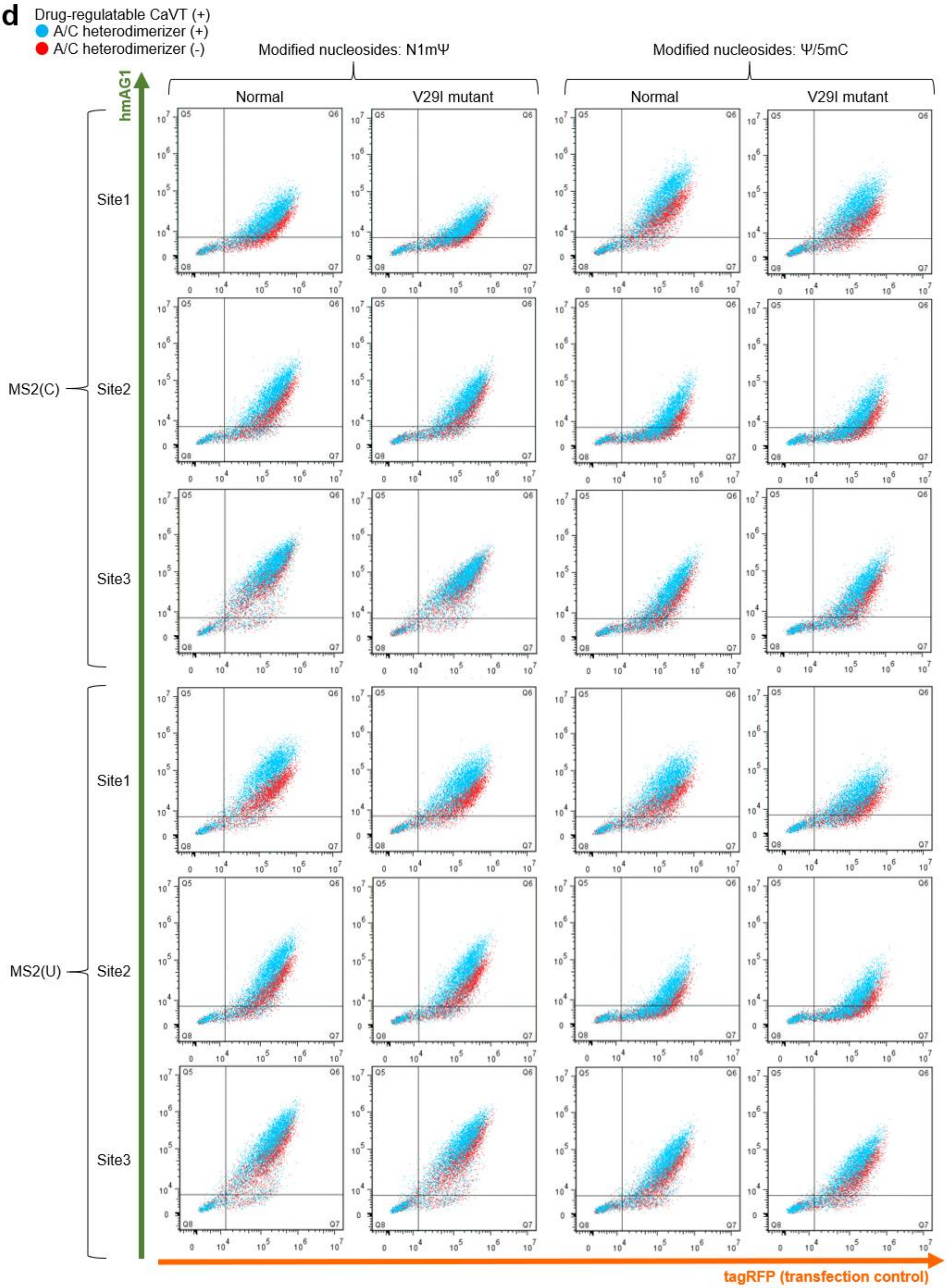
d
Drug-regulatable CaVT (+)
A/C heterodimerizer (+)
A/C heterodimerizer (-)
Modified nucleosides: N1mΨ
Modified nucleosides: Ψ/5mC
Normal
V29I mutant
Normal
V29I mutant
hmAG1
MS2(C)
MS2(U)
Site1
Site2
Site3
Site1
Site2
Site3
Q5
Q6
Q7
Q8
tagRFP (transfection control)

Supplementary Figure 12 | Representative two-dimensional dot plots showing the effects of drug-regulatable CaVT on the translation of target mRNA variants.

HeLa cells were co-transfected with each target hmAG1 mRNA variant, DmrC-VPg(FCV) mRNA, and MS2CP-1xDmrA (or MS2CP(V29I)-1xDmrA) mRNA. After the transfection, the cells were cultured in medium containing 0 or 500 nM A/C heterodimerizer, and the fluorescence was measured by a flow cytometer. (a) The fluorescence of cells without DmrC-VPg(FCV) and MS2CP-1xDmrA (or MS2CP(V29I)-1xDmrA). (b) The fluorescence of cells cultured in medium containing 500 nM A/C heterodimerizer. (c) The fluorescence of cells cultured in medium without A/C heterodimerizer. (d) The superimpositions of (b) (shown as cyan) and (c) (shown as red). Details of the transfection conditions are shown in the supplementary methods.

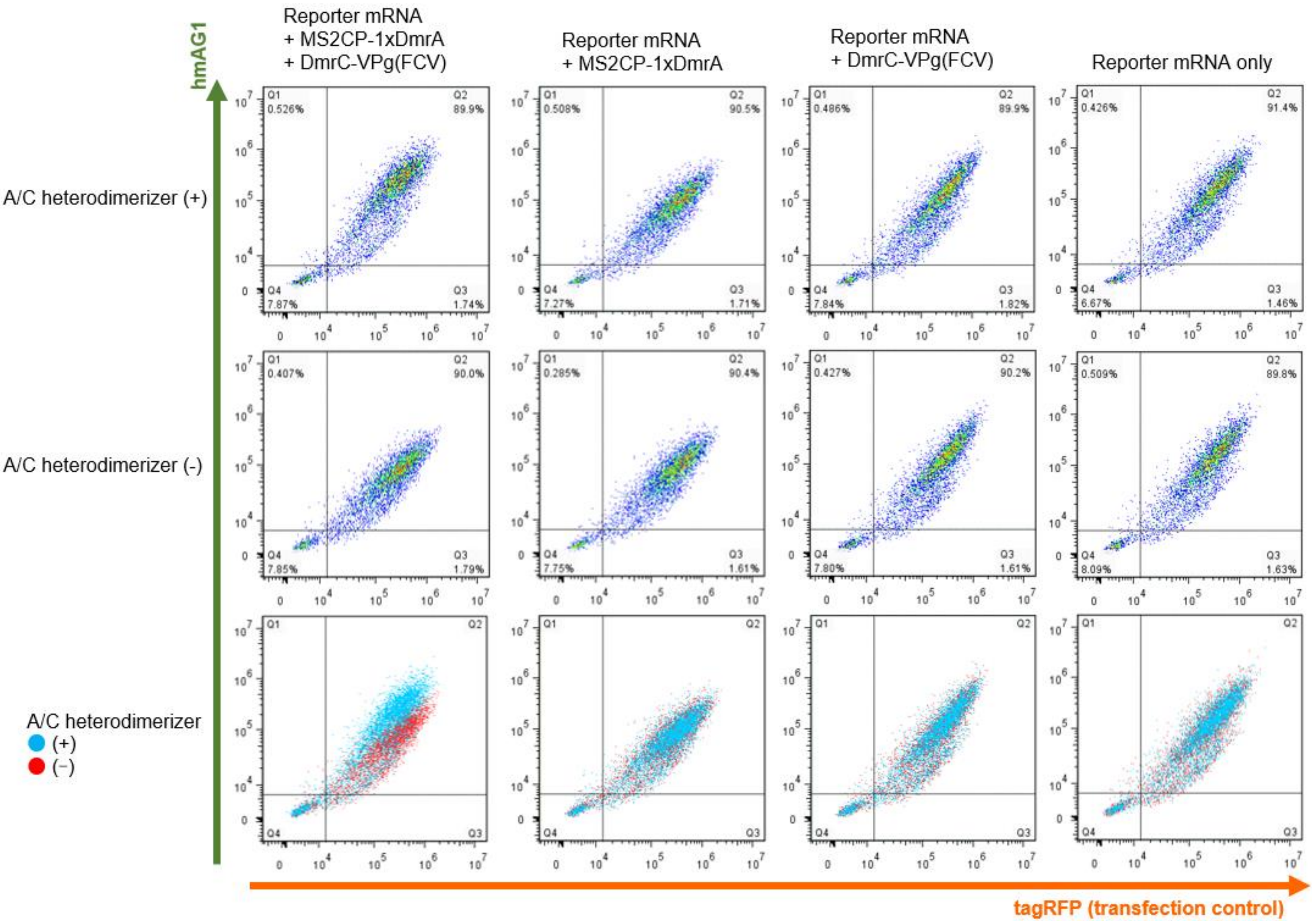

Supplementary Figure 13 | Representative two-dimensional dot plots showing MS2CP-1xDmrA and DmrC-VPg(FCV) dependency of A/C heterodimerizer-mediated translational activation.

HeLa cells were co-transfected with tagRFP mRNA, 1xMS2(U)site1-hmAG1 mRNA, and the mRNAs to express indicated components of the drug-regulatable CaVT. After the transfection, the cells were cultured in medium containing 0 or 500 nM A/C heterodimerizer, and the fluorescence was measured by a flow cytometer. The fluorescence of the cells cultured in medium containing 500 or 0 nM A/C heterodimerizer are shown in the top and middle rows, respectively. The superimpositions of them are shown in the bottom row. Details of the transfection conditions are shown in the supplementary methods.

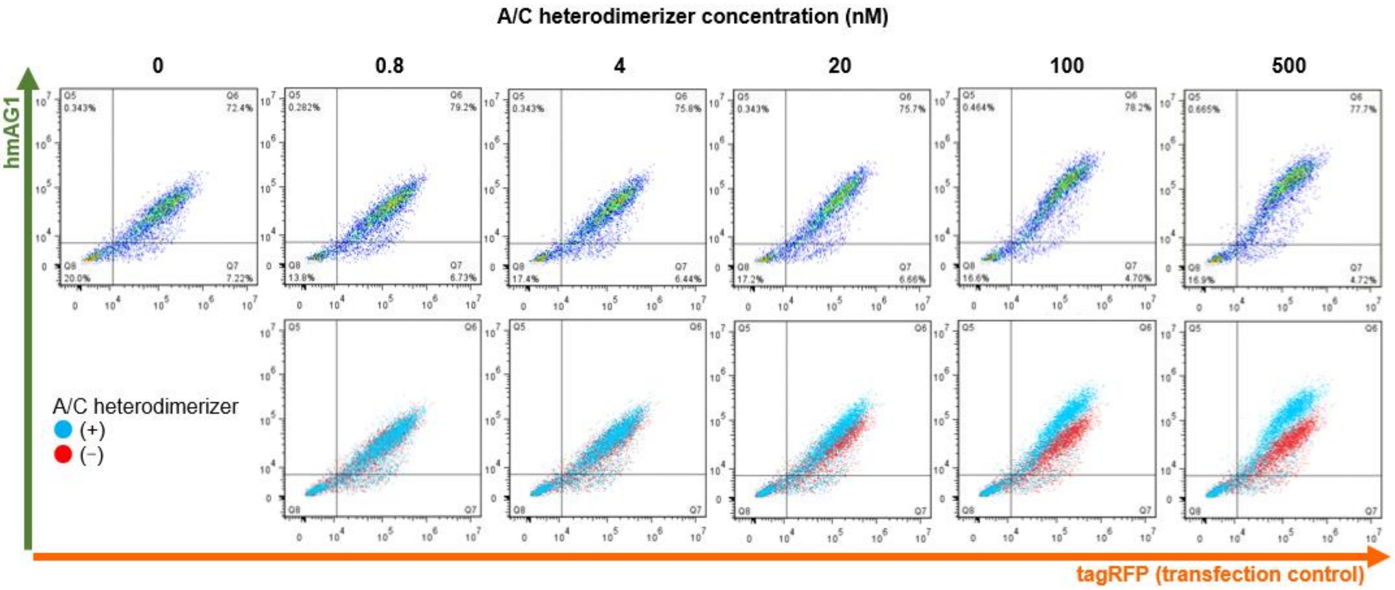

Supplementary Figure 14 | Representative two-dimensional dot plots showing the dose-dependency of drug-regulatable CaVT.

HeLa cells were co-transfected with 1xMS2(C)-hmAG1, DmrC-VPg(FCV), and MS2CP-1xDmrA mRNAs. After the transfection, the cells were cultured in medium containing the indicated concentration of A/C heterodimerizer, and the fluorescence was measured by a flow cytometer. Histograms of the hmAG1 (+)/tagRFP (+) population are shown in Figure 8e. Details of the transfection conditions are shown in the supplementary methods.

Supplementary Table 1 | Exact P values of Figure 5b-c; 6b; 7b, 7d

Figure No.	Sample Name	P value
5b	No Bax mRNA/+ CaVT (15 ng)	0.999
	No Bax mRNA/+ CaVT (30 ng)	0.113
	No Bax mRNA/+ MS2CP (30 ng)	0.115
	1xMS2(U)site2-Bax/No CaVT or MS2CP	< 0.001
	1xMS2(U)site2-Bax/+ CaVT (15 ng)	< 0.001
	1xMS2(U)site2-Bax/+ CaVT (30 ng)	< 0.001
	1xMS2(U)site2-Bax/+ MS2CP (30 ng)	< 0.001
	1xMS2(U)site2-Bax + 2xScMS2(C)-BclxL/No CaVT or MS2CP	0.981
	1xMS2(U)site2-Bax + 2xScMS2(C)-BclxL/+ CaVT (15 ng)	< 0.001
	1xMS2(U)site2-Bax + 2xScMS2(C)-BclxL/+ CaVT (30 ng)	< 0.001
	1xMS2(U)site2-Bax + 2xScMS2(C)-BclxL/+ MS2CP (30 ng)	< 0.001
5c (Annexin V positive)	Bax mRNA with ARCA (Positive control)	< 0.001
	1xMS2(U)site2-Bax/No CaVT	< 0.001
	1xMS2(U)site2-Bax/+ CaVT	< 0.001
	1xMS2(U)site2-Bax + 2xScMS2(C)-BclxL/No CaVT	0.277
	1xMS2(U)site2-Bax + 2xScMS2(C)-BclxL/+ CaVT	< 0.001

5c (Annexin V and SYTOX Red positive)	Bax mRNA with ARCA (Positive control)	< 0.001
	1xMS2(U)site2-Bax/No CaVT	0.009
	1xMS2(U)site2-Bax/+ CaVT	< 0.001
	1xMS2(U)site2-Bax + 2xScMS2(C)-BclxL/No CaVT	0.980
	1xMS2(U)site2-Bax + 2xScMS2(C)-BclxL/+ CaVT	< 0.001
6b	No sgRNA (Negative control)	1.000
	Cas9 mRNA with ARCA (Positive control)	< 0.001
	1xMS2(U)site2-Cas9/No CaVT	< 0.001
	1xMS2(U)site2-Cas9/+ CaVT	< 0.001
	1xMS2(U)site2-Cas9 + 2xScMS2(C)-AcrIIA4 (0.5 ng)/No CaVT	0.998
	1xMS2(U)site2-Cas9 + 2xScMS2(C)-AcrIIA4 (0.5 ng)/+ CaVT	< 0.001
	1xMS2(U)site2-Cas9 + 2xScMS2(C)-AcrIIA4 (1 ng)/No CaVT	1.000
	1xMS2(U)site2-Cas9 + 2xScMS2(C)-AcrIIA4 (1 ng)/+ CaVT	< 0.001
7b (Annexin V positive)	Bax mRNA with ARCA (Positive control)	< 0.001
	1xMS2(U)site2-Bax + 2xScMS2(C)-BclxL<br>/+ miR-302a-5p-responsive CaVT/+ miR-302a-5p mimic	0.987
	1xMS2(U)site2-Bax + 2xScMS2(C)-BclxL<br>/+ miR-302a-5p-responsive CaVT/+ Control mimic	< 0.001
	1xMS2(U)site2-Bax + 2xScMS2(C)-BclxL<br>/+ CaVT/+ miR-302a-5p mimic	< 0.001
	1xMS2(U)site2-Bax + 2xScMS2(C)-BclxL<br>/+ CaVT/+ Control mimic	< 0.001
	1xMS2(U)site2-Bax + 2xScMS2(C)-BclxL<br>/+ miR-21-5p-responsive CaVT/+ miR-21-5p inhibitor	< 0.001
	1xMS2(U)site2-Bax + 2xScMS2(C)-BclxL<br>/+ miR-21-5p-responsive CaVT/+ Control inhibitor	0.999
	1xMS2(U)site2-Bax + 2xScMS2(C)-BclxL<br>/CaVT/+ miR-21-5p inhibitor	< 0.001
	1xMS2(U)site2-Bax + 2xScMS2(C)-BclxL<br>/CaVT/+ Control inhibitor	< 0.001
7b (Annexin V and SYTOX Red positive)	Bax mRNA with ARCA (Positive control)	< 0.001
	1xMS2(U)site2-Bax + 2xScMS2(C)-BclxL<br>/+ miR-302a-5p-responsive CaVT/+ miR-302a-5p mimic	0.979
	1xMS2(U)site2-Bax + 2xScMS2(C)-BclxL<br>/+ miR-302a-5p-responsive CaVT/+ Control mimic	0.007
	1xMS2(U)site2-Bax + 2xScMS2(C)-BclxL<br>/+ CaVT/+ miR-302a-5p mimic	0.006
	1xMS2(U)site2-Bax + 2xScMS2(C)-BclxL<br>/+ CaVT/+ Control mimic	0.017
	1xMS2(U)site2-Bax + 2xScMS2(C)-BclxL<br>/+ miR-21-5p-responsive CaVT/+ miR-21-5p inhibitor	0.025

	1xMS2(U)site2-Bax + 2xScMS2(C)-BclxL /+ miR-21-5p-responsive CaVT/+ Control inhibitor	1.000
	1xMS2(U)site2-Bax + 2xScMS2(C)-BclxL /CaVT/+ miR-21-5p inhibitor	0.035
	1xMS2(U)site2-Bax + 2xScMS2(C)-BclxL /CaVT/+ Control inhibitor	0.012
7d (HeLa cells)	No sgRNA (Negative control)	0.997
	Cas9 mRNA with ARCA (Positive control)	< 0.001
	1xMS2(U)site2-Cas9 + 2xScMS2(C)-AcrIIA4 (0.5 ng) /No CaVT	0.993
	1xMS2(U)site2-Cas9 + 2xScMS2(C)-AcrIIA4 (0.5 ng) /+ miR-302a-5p-responsive CaVT	< 0.001
	1xMS2(U)site2-Cas9 + 2xScMS2(C)-AcrIIA4 (0.5 ng) /+ miR-21-5p-responsive CaVT	0.917
	1xMS2(U)site2-Cas9 + 2xScMS2(C)-AcrIIA4 (0.5 ng) /+ CaVT	< 0.001
7d (iPS cells)	No sgRNA (Negative control)	1.000
	Cas9 mRNA with ARCA (Positive control)	< 0.001
	1xMS2(U)site2-Cas9 + 2xScMS2(C)-AcrIIA4 (0.5 ng) /No CaVT	1.000
	1xMS2(U)site2-Cas9 + 2xScMS2(C)-AcrIIA4 (0.5 ng) /+ miR-302a-5p-responsive CaVT	0.081
	1xMS2(U)site2-Cas9 + 2xScMS2(C)-AcrIIA4 (0.5 ng) /+ miR-21-5p-responsive CaVT	< 0.001
	1xMS2(U)site2-Cas9 + 2xScMS2(C)-AcrIIA4 (0.5 ng) /+ CaVT	< 0.001

Supplementary Methods

Detailed procedures of the pDNA construction

pDNA name	Cloning method	Vector	Insert
pcDNA3.1-MS2CP-VPg(FCV)	Ligation high ver.2 (Toyobo)	BamHI + KpnI cut pcDNA3.1-L7Ae-VPg(FCV)	BamHI + KpnI cut pcDNA3.1-MS2CP-hPABPc1
pcDNA3.1-MS2CP-VPg(NV-GI)	Ligation high ver.2	BamHI + KpnI cut pcDNA3.1-L7Ae-VPg(NV-GI)	BamHI + KpnI cut pcDNA3.1-MS2CP-hPABPc1
pcDNA3.1-MS2CP(V29I)-VPg(FCV)	KOD -Plus-Mutagenesis Kit (Toyobo)	PCR product (Template: pcDNA3.1-MS2CP-VPg(FCV), Forward primer: ATCGCTGAATGGATCAGCTCTAACTC, Reverse primer: CCCGTTAGCGAAGTTGCTTGG)	-
pcDNA3.1-MS2CP-2xDmrA	In-Fusion HD (Takara Bio)	AgeI + BamHI cut pcDNA3.1-MS2CP-VPg(FCV)	PCR product (Template: pHet-Mem1 (Takara Bio), Forward primer: CGGCATCTACGGATCCTCTAGAGGAGTGCAGGT, Reverse primer: CAGTTGGAACACCGGTCTCCAGCTTCAGCAGCT)
pcDNA3.1-MS2CP(V29I)-2xDmrA	In-Fusion HD	AgeI + BamHI cut pcDNA3.1-MS2CP(V29I)-VPg(FCV)	PCR product (Template: pHet-Mem1 (Takara Bio), Forward primer: CGGCATCTACGGATCCTCTAGAGGAGTGCAGGT, Reverse primer: CAGTTGGAACACCGGTCTCCAGCTTCAGCAGCT)
pcDNA3.1-DmrC-VPg(FCV)	In-Fusion HD	BamHI + KpnI cut pcDNA3.1-MS2CP-VPg(FCV)	PCR product (Template: pHet-1 (Takara Bio), Forward primer: TTAAACTTAAGCTTGGTACCGCCACCATGGCTTCTAGA, Reverse primer:

			TGCCCTTGGCGGATCCCTTTGAGATTCGTCGGAAC)
PB-TRE-hmAG1	In-Fusion HD	NotI cut PB-TRE-IRES-hmAG1[1]	PCR product (Template: PB-TRE-IRES-hmAG1, Forward primer:GCAGGCTCCGCGGCCACCATGGTGAGCGTGATCAAGCCCGAGA, Reverse primer: ACCGCTAGTGCGGCCGCTG)
pcDNA3.1-L7Ae-VPg(FCV)	In-Fusion HD	AgeI + BamHI cut pcDNA3.1-L7Ae-hPABPc1	VPg(FCV) gene (GeneArt strings artificial gene synthesis, Thermo Fisher Scientific)
pcDNA3.1-L7Ae-VPg(NV-GI)	In-Fusion HD	AgeI + BamHI cut pcDNA3.1-L7Ae-hPABPc1	VPg(NV-GI) gene (GeneArt strings artificial gene synthesis, Thermo Fisher Scientific)
pcDNA3.1-L7Ae-hPABPc1	In-Fusion HD	BamHI cut pcDNA3.1-hPABPc1-DYK (GenScript)	PCR product (Template: pKloop-L7Ae-ECFP-IRES2-DsRed-Express, Forward primer: TACCGAGCTCGGATCGCCACCATGTACGTGAGA, Reverse primer: TCATGGTGGCGGATCCCTTCTGAAGGCCTTTAATCT)
pcDNA3.1-MS2CP-hPABPc1	In-Fusion HD	BamHI cut pcDNA3.1-hPABPc1-DYK (GenScript)	BamHI + BglII cut PCR product (Template: pCTp-MS2CP, Forward primer: aaAGATCTgccaccatgGCTTCTAACTTT, Reverse primer: gggGGATCCGTAGATGCCGGAGTTGGCCGCGATG)

Full sequences of template DNAs for in vitro transcription

・MS2CP

Template pDNA for PCR: pcDNA3.1-MS2CP-VPg(FCV)

Primers for 1st PCR: HNC-365, HNC-379

Primers for 2nd PCR: HNC-242, HNC-396, KEC-62, 3'UTR oligo DNA

T7 promoter, Kozak sequence (including start codon), MS2CP gene

[CAGTGAATTGTAATACGACTCACTATAGGGCGAATTAAGAGAGAAAAGAAGAGTAAGAAGAAATATA
AGACACCGGTCGCCACCATGGCTTCTAACTTTACTCAGTTCGTTCTCGTCGACAATGGCGGAACTGG
CGACGTGACTGTCGCCCCAAGCAACTTCGCTAACGGGGTCGCTGAATGGATCAGCTCTAACTCGCG
ATCACAGGCTTACAAAGTAACCTGTAGCGTTCGTCAGAGCTCTGCGCAGAATCGCAAATACACCATC
AAAGTCGAGGTGCCTAAAGGCGCATGGAGGTCTTACTTAAATATGGAACTAACCATTCCAATTTTCG
CCACGAATTCCGACTGCGAGCTTATTGTTAAGGCAATGCAAGGTCTCCTAAAAGATGGAAACCCGAT
TCCCTCGGCCATCGCGGCCAACTCCGGCATCTACTGAATCTAGACCTTCTGCGGGGCTTGCCTTCT
GGCCATGCCCTTCTTCTCTCCCTTGCACCTGTACCTCTTGGTCTTTGAATAAAGCCTGAGTAGGAAA
AAAAAAAAAAAAAAAAAAAAAAAAAAAAAAAAAAAAAAAAAAAAAAAAAAAAAAAAAAAAAAAAAAAAAAA
AAAAAAAAAAAAAAAAAAAAAAAAAAAAAAAAAAAAAAAAAAAAAAA]

・VPg(FCV)

Template pDNA for PCR: pcDNA3.1-MS2CP-VPg(FCV)

Primers for 1st PCR: HNC-266, HNC-372

Primers for 2nd PCR: HNC-242, HNC-396, KEC-62, 3'UTR oligo DNA

T7 promoter, Kozak sequence (including start codon), VPg(FCV) gene, DYK-tag gene

[CAGTGAATTGTAATACGACTCACTATAGGGCGAATTAAGAGAGAAAAGAAGAGTAAGAAGAAATATA
AGACACCGGTCGCCACCATGGCCAAGGGCAAGACCAAGAGCAAAGTGGGCCCCTACAGAGGCAGAG
GCGTGGCCCTTACAGACGACGAGTATGACGAATGGCGCGAGCACAACGCCACCAGAAAGCTGGATC
TGAGCGTGGAAGATTTCCTGATGCTGCGGCACAGAGCCGCTCTGGGAGCTGATGATGCCGACGCCG
TGAAGTTCAGATCCTGGTGGAACAGCAGAAGCCGGCTGGCCGACGATTACGAGGATGTGACCGTGA
TCGGCAAAGGCGGCGTGAAGCACGAGAAGATCCGGACCAATACTCTGAGAGCCGTGGACAGAGGCT
ACGACGTGTCCTTCGCTGAAGAAACCGGTGTTCCAACTGTTGATTACAAGGATGACGACGATAAGTG
AATCTAGACCTTCTGCGGGGCTTGCCTTCTGGCCATGCCCTTCTTCTCTCCCTTGCACCTGTACCTC
TTGGTCTTTGAATAAAGCCTGAGTAGGAAAAAAAAAAAAAAAAAAAAAAAAAAAAAAAAAAAAAAAAAA
AAAAAAAAAAAAAAAAAAAAAAAAAAAAAAAAAAAAAAAAAAAAAAAAAAAAAAAAAAAAAAAAAAAAAAA
AAAAAAAA]

・VPg(NV-GI)

Template pDNA for PCR: pcDNA3.1-MS2CP-VPg(NV-GI)

Primers for 1st PCR: HNC-266, HNC-373

Primers for 2nd PCR: HNC-242, HNC-396, KEC-62, 3'UTR oligo DNA

T7 promoter, Kozak sequence (including start codon), VPg(NV-GI) gene, DYK-tag gene

[CAGTGAATTGTAATACGACTCACTATAGGGCGAATTAAGAGAGAAAAGAAGAGTAAGAAGAAATATA
AGACACCGGTCGCCACCATGGGCAAGAACAAGGGCAAGACCAAGAAAGGCAGAGGCCGGAAGAACA

ACTACAACGCCTTCAGCAGACGGGGCCTGAGCGACGAGGAATACGAAGAGTACAAGAAGATCCGGG
AAGAGAAGAACGGCAACTACAGCATCCAAGAGTACCTGGAAGATCGGCAGCGCTACGAAGAGGAACT
GGCCGAAGTTCAGGCTGGCGGAGATGGCGGAATTGGCGAGACAGAGATGGAAATCCGGCACCGGG
TGTTCTACAAGTCCAAGAGCAAGAAGCACCAGCAAGAGCAGCGGAGACAGCTGGGACTCGTGACAG
GCAGCGACATCAGAAAGCGGAAGCCCATCGATTGGACCCCTCCAAAGAACGAGTGGGCCGACGACG
ATAGAGAGGTGGACTACAACGAGAAGATCAACTTCGAAACCGGTGTTCCAACTGTTGATTACAAGGA
TGACGACGATAAGTGAATCTAGACCTTCTGCGGGGCTTGCCTTCTGGCCATGCCCTTCTTCTCTCCC
TTGCACCTGTACCTCTTGGTCTTTGAATAAAGCCTGAGTAGGAAAAAAAAAAAAAAAAAAAAAAAAAA
AAAAAAAAAAAAAAAAAAAAAAAAAAAAAAAAAAAAAAAAAAAAAAAAAAAAAAAAAAAAAAAAAAAAAAAAAAAA
AAAAAAAAAAAAAAAAAAAAAAAA]

・**CaVT (MS2CP-VPg(FCV))**

Template pDNA for PCR: pcDNA3.1-MS2CP-VPg(FCV)

Primers for 1st PCR: HNC-365, HNC-266

Primers for 2nd PCR: HNC-242, HNC-396, KEC-62, 3'UTR oligo DNA

T7 promoter, Kozak sequence (including start codon), MS2CP gene, VPg(FCV) gene, DYK-tag gene

[CAGTGAATTGTAATACGACTCACTATAGGGCGAATTAAGAGAGAAAAGAAGAGTAAGAAGAAATATA
AGACACCGGTCGCCACCatgGCTTCTAACTTTACTCAGTTCGTTCTCGTCGACAATGGCGGAACTGGC
GACGTGACTGTCGCCCCAAGCAACTTCGCTAACGGGGTCGCTGAATGGATCAGCTCTAACTCGCGA
TCACAGGCTTACAAAGTAACCTGTAGCGTTCGTCAGAGCTCTGCGCAGAATCGCAAATACACCATCA
AAGTCGAGGTGCCTAAAGGCGCATGGAGGTCTTACTTAAATATGGAACTAACCATTCCAATTTTCGC
CACGAATTCCGACTGCGAGCTTATTGTTAAGGCAATGCAAGGTCTCCTAAAAGATGGAAACCCGATT
CCCTCGGCCATCGCGGCCAACTCCGGCATCTACGGATCCGCCAAGGGCAAGACCAAGAGCAAAGTG
GGCCCCTACAGAGGCAGAGGCGTGGCCCTTACAGACGACGAGTATGACGAATGGCGCGAGCACAAC
GCCACCAGAAAGCTGGATCTGAGCGTGGAAGATTTCCTGATGCTGCGGCACAGAGCCGCTCTGGGA
GCTGATGATGCCGACGCCGTGAAGTTCAGATCCTGGTGGAACAGCAGAAGCCGGCTGGCCGACGAT
TACGAGGATGTGACCGTGATCGGCAAAGGCGGCGTGAAGCACGAGAAGATCCGGACCAATACTCTG
AGAGCCGTGGACAGAGGCTACGACGTGTCCTTCGCTGAAGAAACCGGTGTTCCAACTGTTGATTAC
AAGGATGACGACGATAAGTGAATCTAGACCTTCTGCGGGGCTTGCCTTCTGGCCATGCCCTTCTTC
TCTCCCTTGCACCTGTACCTCTTGGTCTTTGAATAAAGCCTGAGTAGGAAAAAAAAAAAAAAAAAAAAA
AAAAAAAAAAAAAAAAAAAAAAAAAAAAAAAAAAAAAAAAAAAAAAAAAAAAAAAAAAAAAAAAAAAAAAAAAAAA
AAAAAAAAAAAAAAAAAAAAAAAAAAAAAA]

・**MS2CP-VPg(NV-GI)**

Template pDNA for PCR: pcDNA3.1-MS2CP-VPg(NV-GI)

Primers for 1st PCR: HNC-365, HNC-266

Primers for 2nd PCR: HNC-242, HNC-396, KEC-62, 3'UTR oligo DNA

T7 promoter, Kozak sequence (including start codon), MS2CP gene, VPg(NV-GI) gene, DYK-tag gene

[CAGTGAATTGTAATACGACTCACTATAGGGCGAATTAAGAGAGAAAAGAAGAGTAAGAAGAAATATA
AGACACCGGTCgccaccatgGCTTCTAACTTTACTCAGTTCGTTCTCGTCGACAATGGCGGAACTGGCGA
CGTGACTGTCGCCCCAAGCAACTTCGCTAACGGGGTCGCTGAATGGATCAGCTCTAACTCGCGATCA
CAGGCTTACAAAGTAACCTGTAGCGTTCGTCAGAGCTCTGCGCAGAATCGCAAATACACCATCAAAG

TCGAGGTGCCTAAAGGCGCATGGAGGTCTTACTTAAATATGGAACTAACCATTCCAATTTTCGCCAC
GAATTCCGACTGCGAGCTTATTGTTAAGGCAATGCAAGGTCTCCTAAAAGATGGAAACCCGATTCCC
TCGGCCATCGCGGCCAACTCCGGCATCTACGGATCCGGCAAGAACAAGGGCAAGACCAAGAAAGGC
AGAGGCCGGAAGAACAACTACAACGCCTTCAGCAGACGGGGCCTGAGCGACGAGGAATACGAAGAG
TACAAGAAGATCCGGGAAGAGAAGAACGGCAACTACAGCATCCAAGAGTACCTGGAAGATCGGCAGC
GCTACGAAGAGGAACTGGCCGAAGTTCAGGCTGGCGGAGATGGCGGAATTGGCGAGACAGAGATG
GAAATCCGGCACCGGGTGTTCTACAAGTCCAAGAGCAAGAAGCACCAGCAAGAGCAGCGGAGACAG
CTGGGACTCGTGACAGGCAGCGACATCAGAAAGCGGAAGCCCATCGATTGGACCCCTCCAAAGAAC
GAGTGGGCCGACGACGATAGAGAGGTGGACTACAACGAGAAGATCAACTTCGAAACCGGTGTTCCA
ACTGTTGATTACAAGGATGACGACGATAAGTGAATCTAGACCTTCTGCGGGGCTTGCCTTCTGGCC
ATGCCCTTCTTCTCTCCCTTGCACCTGTACCTCTTGGTCTTTGAATAAAGCCTGAGTAGGAAAAAA
AAAAAAAAAAAAAAAAAAAAAAAAAAAAAAAAAAAAAAAAAAAAAAAAAAAAAAAAAAAAAAAAAAAAAAA
AAAAAAAAAAAAAAAAAAAAAAAAAAAAAAAAAAAAAAAAAAA]

・CaVT V29I mutant (MS2CP(V29I)-VPg)

Template pDNA for PCR: pcDNA3.1-MS2CP(V29I)-VPg(FCV)

Primers for 1st PCR: HNC-365, HNC-266

Primers for 2nd PCR: HNC-242, HNC-396, KEC-62, 3'UTR oligo DNA

T7 promoter, Kozak sequence (including start codon), MS2CP(V29I) gene, VPg(FCV) gene, DYK-tag gene

[CAGTGAATTGTAATACGACTCACTATAGGGCGAATTAAGAGAGAAAAGAAGAGTAAGAAGAAATATA
AGACACCGGTCGCCACCatgGCTTCTAACTTTACTCAGTTCGTTCTCGTCGACAATGGCGGAACTGGC
GACGTGACTGTCGCCCCAAGCAACTTCGCTAACGGGATCGCTGAATGGATCAGCTCTAACTCGCGAT
CACAGGCTTACAAAGTAACCTGTAGCGTTCGTCAGAGCTCTGCGCAGAATCGCAAATACACCATCAA
AGTCGAGGTGCCTAAAGGCGCATGGAGGTCTTACTTAAATATGGAACTAACCATTCCAATTTTCGCC
ACGAATTCCGACTGCGAGCTTATTGTTAAGGCAATGCAAGGTCTCCTAAAAGATGGAAACCCGATTC
CCTCGGCCATCGCGGCCAACTCCGGCATCTACGGATCCGCCAAGGGCAAGACCAAGAGCAAAGTGG
GCCCCTACAGAGGCAGAGGCGTGGCCCTTACAGACGACGAGTATGACGAATGGCGCGAGCACAACG
CCACCAGAAAGCTGGATCTGAGCGTGGAAGATTTCCTGATGCTGCGGCACAGAGCCGCTCTGGGAG
CTGATGATGCCGACGCCGTGAAGTTCAGATCCTGGTGGAACAGCAGAAGCCGGCTGGCCGACGATT
ACGAGGATGTGACCGTGATCGGCAAAGGCGGCGTGAAGCACGAGAAGATCCGGACCAATACTCTGA
GAGCCGTGGACAGAGGCTACGACGTGTCCTTCGCTGAAGAAACCGGTGTTCCAACTGTTGATTACA
AGGATGACGACGATAAGTGAATCTAGACCTTCTGCGGGGCTTGCCTTCTGGCCATGCCCTTCTTCT
CTCCCTTGCACCTGTACCTCTTGGTCTTTGAATAAAGCCTGAGTAGGAAAAAAAAAAAAAAAAAAAAA
AAAAAAAAAAAAAAAAAAAAAAAAAAAAAAAAAAAAAAAAAAAAAAAAAAAAAAAAAAAAAAAAAAAAAAA
AAAAAAAAAAAAAAAAAAAAAAAAAAAAA]

・hsa-miR-21-5p-responsive CaVT

Template pDNA for PCR: pcDNA3.1-MS2CP-VPg(FCV)

Primers for 1st PCR: HNC-401, HNC-266

Primers for 2nd PCR: HNC-378, HNC-396, 3'UTR oligo DNA

T7 promoter, hsa-miR-21-5p target site, Kozak sequence (including start codon), MS2CP gene, VPg(FCV) gene, DYK-tag gene

[CAGTGAATTGTAATACGACTCACTATAGGGCGAATTAAGAGAGAAAAGAAGAGTAAGAAGAAATATA
AGACACCGGTCTCAACATCAGTCTGATAAGCTAGCCACCatgGCTTCTAACTTTACTCAGTTCGTTCT
CGTCGACAATGGCGGAACTGGCGACGTGACTGTCGCCCCAAGCAACTTCGCTAACGGGGTCGCTGA
ATGGATCAGCTCTAACTCGCGATCACAGGCTTACAAAGTAACCTGTAGCGTTCGTCAGAGCTCTGCG
CAGAATCGCAAATACACCATCAAAGTCGAGGTGCCTAAAGGCGCATGGAGGTCTTACTTAAATATGG
AACTAACCATTCCAATTTTCGCCACGAATTCCGACTGCGAGCTTATTGTTAAGGCAATGCAAGGTCT
CCTAAAAGATGGAAACCCGATTCCCTCGGCCATCGCGGCCAACTCCGGCATCTACGGATCCGCCAAG
GGCAAGACCAAGAGCAAAGTGGGCCCCTACAGAGGCAGAGGCGTGGCCCTTACAGACGACGAGTAT
GACGAATGGCGCGAGCACAACGCCACCAGAAAGCTGGATCTGAGCGTGGAAGATTTCCTGATGCTG
CGGCACAGAGCCGCTCTGGGAGCTGATGATGCCGACGCCGTGAAGTTCAGATCCTGGTGGAACAGC
AGAAGCCGGCTGGCCGACGATTACGAGGATGTGACCGTGATCGGCAAAGGCGGCGTGAAGCACGAG
AAGATCCGGACCAATACTCTGAGAGCCGTGGACAGAGGCTACGACGTGTCCTTCGCTGAAGAAACC
GGTGTTCCAACTGTTGATTACAAGGATGACGACGATAAGTGAATCTAGACCTTCTGCGGGGCTTGC
CTTCTGGCCATGCCCTTCTTCTCTCCCTTGCACCTGTACCTCTTGGTCTTTGAATAAAGCCTGAGTA
GGAAAAAAAAAAAAAAAAAAAAAAAAAAAAAAAAAAAAAAAAAAAAAAAAAAAAAAAAAAAAAAAAAAAA
AAAAAAAAAAAAAAAAAAAAAAAAAAAAAAAAAAAAAAAAAAAAAAAAAAAA]

・hsa-miR-302a-5p-responsive CaVT

Template pDNA for PCR: pcDNA3.1-MS2CP-VPg(FCV)

Primers for 1st PCR: HNC-475, HNC-266

Primers for 2nd PCR: HNC-378, HNC-396, 3'UTR oligo DNA

T7 promoter, hsa-miR-302a-5p target site, Kozak sequence (including start codon), MS2CP gene, VPg(FCV) gene, DYK-tag gene

[CAGTGAATTGTAATACGACTCACTATAGGGCGAATTAAGAGAGAAAAGAAGAGTAAGAAGAAATATA
AGACACCGGTCAGCAAGTACATCCACGTTTAAGTGCCACCatgGCTTCTAACTTTACTCAGTTCGTTC
TCGTCGACAATGGCGGAACTGGCGACGTGACTGTCGCCCCAAGCAACTTCGCTAACGGGGTCGCTG
AATGGATCAGCTCTAACTCGCGATCACAGGCTTACAAAGTAACCTGTAGCGTTCGTCAGAGCTCTGC
GCAGAATCGCAAATACACCATCAAAGTCGAGGTGCCTAAAGGCGCATGGAGGTCTTACTTAAATATG
GAACTAACCATTCCAATTTTCGCCACGAATTCCGACTGCGAGCTTATTGTTAAGGCAATGCAAGGTC
TCCTAAAAGATGGAAACCCGATTCCCTCGGCCATCGCGGCCAACTCCGGCATCTACGGATCCGCCAA
GGGCAAGACCAAGAGCAAAGTGGGCCCCTACAGAGGCAGAGGCGTGGCCCTTACAGACGACGAGTA
TGACGAATGGCGCGAGCACAACGCCACCAGAAAGCTGGATCTGAGCGTGGAAGATTTCCTGATGCT
GCGGCACAGAGCCGCTCTGGGAGCTGATGATGCCGACGCCGTGAAGTTCAGATCCTGGTGGAACAG
CAGAAGCCGGCTGGCCGACGATTACGAGGATGTGACCGTGATCGGCAAAGGCGGCGTGAAGCACGA
GAAGATCCGGACCAATACTCTGAGAGCCGTGGACAGAGGCTACGACGTGTCCTTCGCTGAAGAAAC
CGGTGTTCCAACTGTTGATTACAAGGATGACGACGATAAGTGAATCTAGACCTTCTGCGGGGCTTG
CCTTCTGGCCATGCCCTTCTTCTCTCCCTTGCACCTGTACCTCTTGGTCTTTGAATAAAGCCTGAGT
AGGAAAAAAAAAAAAAAAAAAAAAAAAAAAAAAAAAAAAAAAAAAAAAAAAAAAAAAAAAAAAAAAAAA
AAAAAAAAAAAAAAAAAAAAAAAAAAAAAAAAAAAAAAAAAAAAAAAAAAAAA]

・1xMS2(C)site1-hmAG1

Template pDNA for PCR: PB-TRE-hmAG1

Primers for 1st PCR: HNC-383, KEC-331

Primers for 2nd PCR: HNC-382, HNC-396, 3'UTR oligo DNA

T7 promoter, MS2-binding motif (C), Kozak sequence (including start codon), hmAG1 gene

[CAGTGAATTGTAATACGACTCACTATAGGGACATGAGGATCACCCATGTCGAATTAAGAGAGAAAA
GAAGAGTAAGAAGAAATATAAGACACCGGTCGCCACCATGGTGAGCGTGATCAAGCCCGAGATGAA
GATCAAGCTGTGCATGAGGGGCACCGTGAACGGCCACAACTTCGTGATCGAGGGCGAGGGCAAGGG
CAACCCCTACGAGGGCACCCAGATCCTGGACCTGAACGTGACCGAGGGCGCCCCCCTGCCCTTCGC
CTACGACATCCTGACCACCGTGTTCCAGTACGGCAACAGGGCCTTCACCAAGTACCCCGCCGACATC
CAGGACTACTTCAAGCAGACCTTCCCCGAGGGCTACCACTGGGAGAGGAGCATGACCTACGAGGAC
CAGGGCATCTGCACCGCCACCAGCAACATCAGCATGAGGGGCGACTGCTTCTTCTACGACATCAGGT
TCGACGGCACCAACTTCCCCCCCAACGGCCCCGTGATGCAGAAGAAGACCCTGAAGTGGGAGCCCA
GCACCGAGAAGATGTACGTGGAGGACGGCGTGCTGAAGGGCGACGTGAACATGAGGCTGCTGCTG
GAGGGCGGCGGCCACTACAGGTGCGACTTCAAGACCACCTACAAGGCCAAGAAGGAGGTGAGGCTG
CCCGACGCCCACAAGATCGACCACAGGATCGAGATCCTGAAGCACGACAAGGACTACAACAAGGTGA
AGCTGTACGAGAACGCCGTGGCCAGGTACTCCATGCTGCCCAGCCAGGCCAAGtgaATCTAGACCTTC
TGCGGGGCTTGCCTTCTGGCCATGCCCTTCTTCTCTCCCTTGCACCTGTACCTCTTGGTCTTTGAAT
AAAGCCTGAGTAGGAAAAAAAAAAAAAAAAAAAAAAAAAAAAAAAAAAAAAAAAAAAAAAAAAAAAAA
AAAAAAAAAAAAAAAAAAAAAAAAAAAAAAAAAAAAAAAAAAAAAAAAAAAAAAAAAAAAAAAAA]

・1xMS2(C)site2-hmAG1

Template pDNA for 1st PCR: pENTR-IRES-hmAG1

Primers for 1st PCR: KEC-330, KEC-331

Primers for 2nd PCR: HNC-242, HNC-396, HNC-446, 3'UTR oligo DNA

T7 promoter, MS2-binding motif (C), Kozak sequence (including start codon), hmAG1 gene

[CAGTGAATTGTAATACGACTCACTATAGGGCGAATTAAGAGAGAAAAGAAGAGTACATGAGGATCA
CCCATGTAAGAAGAAATATAAGACACCGGTCGCCACCATGGTGAGCGTGATCAAGCCCGAGATGAAG
ATCAAGCTGTGCATGAGGGGCACCGTGAACGGCCACAACTTCGTGATCGAGGGCGAGGGCAAGGGC
AACCCCTACGAGGGCACCCAGATCCTGGACCTGAACGTGACCGAGGGCGCCCCCCTGCCCTTCGCC
TACGACATCCTGACCACCGTGTTCCAGTACGGCAACAGGGCCTTCACCAAGTACCCCGCCGACATCC
AGGACTACTTCAAGCAGACCTTCCCCGAGGGCTACCACTGGGAGAGGAGCATGACCTACGAGGACC
AGGGCATCTGCACCGCCACCAGCAACATCAGCATGAGGGGCGACTGCTTCTTCTACGACATCAGGTT
CGACGGCACCAACTTCCCCCCCAACGGCCCCGTGATGCAGAAGAAGACCCTGAAGTGGGAGCCCAG
CACCGAGAAGATGTACGTGGAGGACGGCGTGCTGAAGGGCGACGTGAACATGAGGCTGCTGCTGG
AGGGCGGCGGCCACTACAGGTGCGACTTCAAGACCACCTACAAGGCCAAGAAGGAGGTGAGGCTGC
CCGACGCCCACAAGATCGACCACAGGATCGAGATCCTGAAGCACGACAAGGACTACAACAAGGTGAA
GCTGTACGAGAACGCCGTGGCCAGGTACTCCATGCTGCCCAGCCAGGCCAAGtgaATCTAGACCTTCT
GCGGGGCTTGCCTTCTGGCCATGCCCTTCTTCTCTCCCTTGCACCTGTACCTCTTGGTCTTTGAATA
AAGCCTGAGTAGGAAAAAAAAAAAAAAAAAAAAAAAAAAAAAAAAAAAAAAAAAAAAAAAAAAAAAA
AAAAAAAAAAAAAAAAAAAAAAAAAAAAAAAAAAAAAAAAAAAAAAAAAAAAAAAAAAAAAAA]

・1xMS2(C)site3-hmAG1:

Template pDNA for PCR: PB-TRE-hmAG1

Primers for 1st PCR: HNC-447, KEC-331

Primers for 2nd PCR: HNC-378, HNC-396, 3'UTR oligo DNA

T7 promoter, MS2-binding motif (C), Kozak sequence (including start codon), hmAG1 gene

[CAGTGAATTGTAATACGACTCACTATAGGGCGAATTAAGAGAGAAAAGAAGAGTAAGAAGAAATATA
AGACACCGGTCACATGAGGATCACCCATGTGCCACCATGGTGAGCGTGATCAAGCCCGAGATGAAG
ATCAAGCTGTGCATGAGGGGCACCGTGAACGGCCACAACTTCGTGATCGAGGGCGAGGGCAAGGGC
AACCCCTACGAGGGCACCCAGATCCTGGACCTGAACGTGACCGAGGGCGCCCCCCTGCCCTTCGCC
TACGACATCCTGACCACCGTGTTCCAGTACGGCAACAGGGCCTTCACCAAGTACCCCGCCGACATCC
AGGACTACTTCAAGCAGACCTTCCCCGAGGGCTACCACTGGGAGAGGAGCATGACCTACGAGGACC
AGGGCATCTGCACCGCCACCAGCAACATCAGCATGAGGGGCGACTGCTTCTTCTACGACATCAGGTT
CGACGGCACCAACTTCCCCCCCAACGGCCCCGTGATGCAGAAGAAGACCCTGAAGTGGGAGCCCAG
CACCGAGAAGATGTACGTGGAGGACGGCGTGCTGAAGGGCGACGTGAACATGAGGCTGCTGCTGG
AGGGCGGCGGCCACTACAGGTGCGACTTCAAGACCACCTACAAGGCCAAGAAGGAGGTGAGGCTGC
CCGACGCCCACAAGATCGACCACAGGATCGAGATCCTGAAGCACGACAAGGACTACAACAAGGTGAA
GCTGTACGAGAACGCCGTGGCCAGGTACTCCATGCTGCCCAGCCAGGCCAAGtgaATCTAGACCTTCT
GCGGGGCTTGCCTTCTGGCCATGCCCTTCTTCTCTCCCTTGCACCTGTACCTCTTGGTCTTTGAATA
AAGCCTGAGTAGGAAAAAAAAAAAAAAAAAAAAAAAAAAAAAAAAAAAAAAAAAAAAAAA
AAAAAAAAAAAAAAAAAAAAAAAAAAAAAAAAAAAAAAAAAAAAAAAAAAAAAAAAAAAAAAAA]

・1xMS2(U)site1-hmAG1

Template pDNA for PCR: PB-TRE-hmAG1

Primers for 1st PCR: HNC-383, KEC-331

Primers for 2nd PCR: HNC-408, HNC-396, 3'UTR oligo DNA

T7 promoter, MS2-binding motif (U), Kozak sequence (including start codon), hmAG1 gene

[CAGTGAATTGTAATACGACTCACTATAGGGACATGAGGATTACCCATGTCGAATTAAGAGAGAAAA
GAAGAGTAAGAAGAAATATAAGACACCGGTCGCCACCATGGTGAGCGTGATCAAGCCCGAGATGAA
GATCAAGCTGTGCATGAGGGGCACCGTGAACGGCCACAACTTCGTGATCGAGGGCGAGGGCAAGGG
CAACCCCTACGAGGGCACCCAGATCCTGGACCTGAACGTGACCGAGGGCGCCCCCCTGCCCTTCGC
CTACGACATCCTGACCACCGTGTTCCAGTACGGCAACAGGGCCTTCACCAAGTACCCCGCCGACATC
CAGGACTACTTCAAGCAGACCTTCCCCGAGGGCTACCACTGGGAGAGGAGCATGACCTACGAGGAC
CAGGGCATCTGCACCGCCACCAGCAACATCAGCATGAGGGGCGACTGCTTCTTCTACGACATCAGGT
TCGACGGCACCAACTTCCCCCCCAACGGCCCCGTGATGCAGAAGAAGACCCTGAAGTGGGAGCCCA
GCACCGAGAAGATGTACGTGGAGGACGGCGTGCTGAAGGGCGACGTGAACATGAGGCTGCTGCTG
GAGGGCGGCGGCCACTACAGGTGCGACTTCAAGACCACCTACAAGGCCAAGAAGGAGGTGAGGCTG
CCCGACGCCCACAAGATCGACCACAGGATCGAGATCCTGAAGCACGACAAGGACTACAACAAGGTGA
AGCTGTACGAGAACGCCGTGGCCAGGTACTCCATGCTGCCCAGCCAGGCCAAGtgaATCTAGACCTTC
TGCGGGGCTTGCCTTCTGGCCATGCCCTTCTTCTCTCCCTTGCACCTGTACCTCTTGGTCTTTGAAT
AAAGCCTGAGTAGGAAAAAAAAAAAAAAAAAAAAAAAAAAAAAAAAAAAAAAAAAAAAAA
AAAAAAAAAAAAAAAAAAAAAAAAAAAAAAAAAAAAAAAAAAAAAAAAAAAAAAAAAAAAAAAAA]

・1xMS2(U)site2-hmAG1

Template pDNA for PCR: pENTR-IRES-hmAG1

Primers for 1st PCR: KEC-330, KEC-331

Primers for 2nd PCR: HNC-242, HNC-396, HNC-440, 3'UTR oligo DNA

T7 promoter, MS2-binding motif (U), Kozak sequence (including start codon), hmAG1 gene

[CAGTGAATTGTAATACGACTCACTATAGGGCGAATTAAGAGAGAAAAGAAGAGTACATGAGGATTA
CCCATGTAAGAAGAAATATAAGACACCGGTCGCCACCATGGTGAGCGTGATCAAGCCCGAGATGAAG
ATCAAGCTGTGCATGAGGGGCACCGTGAACGGCCACAACTTCGTGATCGAGGGCGAGGGCAAGGGC
AACCCCTACGAGGGCACCCAGATCCTGGACCTGAACGTGACCGAGGGCGCCCCCCTGCCCTTCGCC
TACGACATCCTGACCACCGTGTTCCAGTACGGCAACAGGGCCTTCACCAAGTACCCCGCCGACATCC
AGGACTACTTCAAGCAGACCTTCCCCGAGGGCTACCACTGGGAGAGGAGCATGACCTACGAGGACC
AGGGCATCTGCACCGCCACCAGCAACATCAGCATGAGGGGCGACTGCTTCTTCTACGACATCAGGTT
CGACGGCACCAACTTCCCCCCCAACGGCCCCGTGATGCAGAAGAAGACCCTGAAGTGGGAGCCCAG
CACCGAGAAGATGTACGTGGAGGACGGCGTGCTGAAGGGCGACGTGAACATGAGGCTGCTGCTGG
AGGGCGGCGGCCACTACAGGTGCGACTTCAAGACCACCTACAAGGCCAAGAAGGAGGTGAGGCTGC
CCGACGCCCACAAGATCGACCACAGGATCGAGATCCTGAAGCACGACAAGGACTACAACAAGGTGAA
GCTGTACGAGAACGCCGTGGCCAGGTACTCCATGCTGCCCAGCCAGGCCAAGtgaATCTAGACCTTCT
GCGGGGCTTGCCTTCTGGCCATGCCCTTCTTCTCTCCCTTGCACCTGTACCTCTTGGTCTTTGAATA
AAGCCTGAGTAGGAAAAAAAAAAAAAAAAAAAAAAAAAAAAAAAAAAAAAAAAAAAAAAAAAAAAAA
AAAAAAAAAAAAAAAAAAAAAAAAAAAAAAAAAAAAAAAAAAAAAAAAAAAAAAAAAAAAAAA]

・1xMS2(U)site3-hmAG1

Template pDNA for PCR: PB-TRE-hmAG1

Primers for 1st PCR: HNC-441, KEC-331

Primers for 2nd PCR: HNC-378, HNC-396, 3'UTR oligo DNA

T7 promoter, MS2-binding motif (U), Kozak sequence (including start codon), hmAG1 gene

[CAGTGAATTGTAATACGACTCACTATAGGGCGAATTAAGAGAGAAAAGAAGAGTAAGAAGAAATATA
AGACACCGGTCACATGAGGATTACCCATGTGCCACCATGGTGAGCGTGATCAAGCCCGAGATGAAG
ATCAAGCTGTGCATGAGGGGCACCGTGAACGGCCACAACTTCGTGATCGAGGGCGAGGGCAAGGGC
AACCCCTACGAGGGCACCCAGATCCTGGACCTGAACGTGACCGAGGGCGCCCCCCTGCCCTTCGCC
TACGACATCCTGACCACCGTGTTCCAGTACGGCAACAGGGCCTTCACCAAGTACCCCGCCGACATCC
AGGACTACTTCAAGCAGACCTTCCCCGAGGGCTACCACTGGGAGAGGAGCATGACCTACGAGGACC
AGGGCATCTGCACCGCCACCAGCAACATCAGCATGAGGGGCGACTGCTTCTTCTACGACATCAGGTT
CGACGGCACCAACTTCCCCCCCAACGGCCCCGTGATGCAGAAGAAGACCCTGAAGTGGGAGCCCAG
CACCGAGAAGATGTACGTGGAGGACGGCGTGCTGAAGGGCGACGTGAACATGAGGCTGCTGCTGG
AGGGCGGCGGCCACTACAGGTGCGACTTCAAGACCACCTACAAGGCCAAGAAGGAGGTGAGGCTGC
CCGACGCCCACAAGATCGACCACAGGATCGAGATCCTGAAGCACGACAAGGACTACAACAAGGTGAA
GCTGTACGAGAACGCCGTGGCCAGGTACTCCATGCTGCCCAGCCAGGCCAAGtgaATCTAGACCTTCT
GCGGGGCTTGCCTTCTGGCCATGCCCTTCTTCTCTCCCTTGCACCTGTACCTCTTGGTCTTTGAATA
AAGCCTGAGTAGGAAAAAAAAAAAAAAAAAAAAAAAAAAAAAAAAAAAAAAAAAAAAAAAAAAAAAA
AAAAAAAAAAAAAAAAAAAAAAAAAAAAAAAAAAAAAAAAAAAAAAAAAAAAAAAAAAAAAAA]

・**2xScMS2(C)-hmAG1**

Template pDNA for PCR: pENTR-IRES-hmAG1

Primers for 1st PCR: KEC-330, KEC-331

Primers for 2nd PCR: HNC-370, HNC-396, 3'UTR oligo DNA

T7 promoter, 2xMS2-binding motif (C) with scaffold, Kozak sequence (including start codon), hmAG1 gene

[CAGTGAATTGTAATACGACTCACTATAGGGTCAGATCCGCTAGCGGATCCgggagcAggtgAGGATCACC
CATcTgccacgagcgAggtgAGGATCACCCATcTcgctcgtgttcccACCGGTCGCCACCATGGTGAGCGTGATCAA
GCCCGAGATGAAGATCAAGCTGTGCATGAGGGGCACCGTGAACGGCCACAACTTCGTGATCGAGGG
CGAGGGCAAGGGCAACCCCTACGAGGGCACCCAGATCCTGGACCTGAACGTGACCGAGGGCGCCCC
CCTGCCCTTCGCCTACGACATCCTGACCACCGTGTTCCAGTACGGCAACAGGGCCTTCACCAAGTAC
CCCGCCGACATCCAGGACTACTTCAAGCAGACCTTCCCCGAGGGCTACCACTGGGAGAGGAGCATG
ACCTACGAGGACCAGGGCATCTGCACCGCCACCAGCAACATCAGCATGAGGGGCGACTGCTTCTTCT
ACGACATCAGGTTCGACGGCACCAACTTCCCCCCCAACGGCCCCGTGATGCAGAAGAAGACCCTGAA
GTGGGAGCCCAGCACCGAGAAGATGTACGTGGAGGACGGCGTGCTGAAGGGCGACGTGAACATGA
GGCTGCTGCTGGAGGGCGGCGGCCACTACAGGTGCGACTTCAAGACCACCTACAAGGCCAAGAAGG
AGGTGAGGCTGCCCGACGCCCACAAGATCGACCACAGGATCGAGATCCTGAAGCACGACAAGGACT
ACAACAAGGTGAAGCTGTACGAGAACGCCGTGGCCAGGTACTCCATGCTGCCCAGCCAGGCCAAGtga
ATCTAGACCTTCTGCGGGGCTTGCCTTCTGGCCATGCCCTTCTTCTCTCCCTTGCACCTGTACCTCT
TGGTCTTTGAATAAAGCCTGAGTAGGAAAAAAAAAAAAAAAAAAAAAAAAAAAAAAAAAAAAAAAAAAA
AAAAAAAAAAAAAAAAAAAAAAAAAAAAAAAAAAAAAAAAAAAAAAAAAAAAAAAAAAAAAAAAAAAAAA
AAAAAAA]

・**hmAG1**

Template pDNA for PCR: pENTR-IRES-hmAG1

Primers for 1st PCR: KEC-330, KEC-331

Primers for 2nd PCR: HNC-242, HNC-396, KEC-62, 3'UTR oligo DNA

T7 promoter, Kozak sequence (including start codon), hmAG1 gene

[CAGTGAATTGTAATACGACTCACTATAGGGCGAATTAAGAGAGAAAAGAAGAGTAAGAAGAAATATA
AGACACCGGTCGCCACCATGGTGAGCGTGATCAAGCCCGAGATGAAGATCAAGCTGTGCATGAGGG
GCACCGTGAACGGCCACAACTTCGTGATCGAGGGCGAGGGCAAGGGCAACCCCTACGAGGGCACCC
AGATCCTGGACCTGAACGTGACCGAGGGCGCCCCCCTGCCCTTCGCCTACGACATCCTGACCACCGT
GTTCCAGTACGGCAACAGGGCCTTCACCAAGTACCCCGCCGACATCCAGGACTACTTCAAGCAGACC
TTCCCCGAGGGCTACCACTGGGAGAGGAGCATGACCTACGAGGACCAGGGCATCTGCACCGCCACC
AGCAACATCAGCATGAGGGGCGACTGCTTCTTCTACGACATCAGGTTCGACGGCACCAACTTCCCCC
CCAACGGCCCCGTGATGCAGAAGAAGACCCTGAAGTGGGAGCCCAGCACCGAGAAGATGTACGTGG
AGGACGGCGTGCTGAAGGGCGACGTGAACATGAGGCTGCTGCTGGAGGGCGGCGGCCACTACAGG
TGCGACTTCAAGACCACCTACAAGGCCAAGAAGGAGGTGAGGCTGCCCGACGCCCACAAGATCGACC
ACAGGATCGAGATCCTGAAGCACGACAAGGACTACAACAAGGTGAAGCTGTACGAGAACGCCGTGG
CCAGGTACTCCATGCTGCCCAGCCAGGCCAAGtgaATCTAGACCTTCTGCGGGGCTTGCCTTCTGGCC
ATGCCCTTCTTCTCTCCCTTGCACCTGTACCTCTTGGTCTTTGAATAAAGCCTGAGTAGGAAAAAAA
AAAAAAAAAAAAAAAAAAAAAAAAAAAAAAAAAAAAAAAAAAAAAAAAAAAAAAAAAAAAAAAAAAAAAA
AAAAAAAAAAAAAAAAAAAAAAAAAAAAAAAAAAAAAAAAAA]

・tagRFP

Template pDNA for PCR: pDuReg2MS-tagRFP-hmAG1(92a-3p)[1]

Primers for 1st PCR: HNC-237, HNC-238

Primers for 2nd PCR: HNC-242, HNC-396, KEC-62, 3'UTR oligo DNA

T7 promoter, Kozak sequence (including start codon), tagRFP gene

[CAGTGAATTGTAATACGACTCACTATAGGGCGAATTAAGAGAGAAAAGAAGAGTAAGAAGAAATATA
AGACACCGGTCGCCACCATGgtgtctaagggcgaagagctgattaaggagaacatgcacatgaagctgtacatggagggcaccgtgaacaacca
ccacttcaagtgcacatccgagggcgaaggcaagccctacgagggcacccagaccatgagaatcaaggtggtcgagggcggccctctccccttcgccttcgacatcc
tggctaccagcttcatgtacggcagcagaaccttcatcaaccacacccagggcatccccgacttctttaagcagtccttccctgagggcttcacatgggagagagtcac
cacatacgaagacgggggcgtgctgaccgctacccaggacaccagcctccaggacggctgcctcatctacaacgtcaagatcagaggggtgaacttcccatccaac
ggccctgtgatgcagaagaaaacactcggctgggaggccaacaccgagatgctgtaccccgctgacggcggcctggaaggcagaagcgacatggccctgaagctc
gtgggcgggggccacctgatctgcaacttcaagaccacatacagatccaagaaacccgctaagaacctcaagatgcccggcgtctactatgtggaccacagactgga
aagaatcaaggaggccgacaaagagacctacgtcgagcagcacgaggtggctgtggccagatactgcgacctccctagcaaactggggcacaaacttaattgaAT
CTAGACCTTCTGCGGGGCTTGCCTTCTGGCCATGCCCTTCTTCTCTCCCTTGCACCTGTACCTCTTG
GTCTTTGAATAAAGCCTGAGTAGGAAAAAAAAAAAAAAAAAAAAAAAAAAAAAAAAAAAAAAAAAAAAAAA
AAAAAAAAAAAAAAAAAAAAAAAAAAAAAAAAAAAAAAAAAAAAAAAAAAAAAAAAAAAAAAAAAAAAAA
AAAAA]

・1xMS2(U)site2-Bax

Template pDNA for PCR: pUC19-hBaxwoT7f

Primers for PCR: HNC-242, HNC-396, HNC-440

T7 promoter, MS2-binding motif (U), Kozak sequence (including start codon), hBax gene

[CAGTGAATTGTAATACGACTCACTATAGGGCGAATTAAGAGAGAAAAGAAGAGTACATGAGGATTA
CCCATGTAAGAAGAAATATAAGACACCGGTCGCCACCATGGACGGGTCCGGGGAGCAGCCCAGAGG
CGGGGGGCCCACCAGCTCTGAGCAGATCATGAAGACAGGGGCCCTTTTGCTTCAGGGTTTCATCCA
GGATCGAGCAGGGCGAATGGGGGGGGAGGCACCCGAGCTGGCCCTGGACCCGGTGCCTCAGGATG
CGTCCACCAAGAAGCTGAGCGAGTGTCTCAAGCGCATCGGGGACGAACTGGACAGTAACATGGAGC
TGCAGAGGATGATTGCCGCCGTGGACACAGACTCCCCCCGAGAGGTCTTTTTCCGAGTGGCAGCTG
ACATGTTTTCTGACGGCAACTTCAACTGGGGCCGGGTTGTCGCCCTTTTCTACTTTGCCAGCAAACT
GGTGCTCAAGGCCCTGTGCACCAAGGTGCCGGAACTGATCAGAACCATCATGGGCTGGACATTGGA
CTTCCTCCGGGAGCGGCTGTTGGGCTGGATCCAAGACCAGGGTGGTTGGGACGGCCTCCTCTCCTA
CTTTGGGACGCCCACGTGGCAGACCGTGACCATCTTTGTGGCGGGAGTGCTCACCGCCTCGCTCAC
CATCTGGAAGAAGATGGGCTGATTCTAGACCTTCTGCGGGGCTTGCCTTCTGGCCATGCCCTTCTT
CTCTCCCTTGCACCTGTACCTCTTGGTCTTTGAATAAAGCCTGAGTAGGAAAAAAAAAAAAAAAAAAA
AAAAAAAAAAAAAAAAAAAAAAAAAAAAAAAAAAAAAAAAAAAAAAAAAAAAAAAAAAAAAAAAAAAAAA
AAAAAAAAAAAAAAAAAAAAAAAAAAAAAAA]

・2xScMS2(C)-BclxL

Template pDNA for PCR: pUC19-BclxLwoT7f

Primers for PCR: HNC-370, HNC-396

T7 promoter, 2xMS2-binding motif (C) with scaffold, Kozak sequence (including start codon), BclxL gene

[CAGTGAATTGTAATACGACTCACTATAGGGTCAGATCCGCTAGCGGATCCgggagcAggtgAGGATCACC CATcTgccacgagcgAggtgAGGATCACCCATcTcgctcgtgttcccACCGGTCGCCACCATGTCTCAGAGcaaccgggag ctggtggttgactttctctcctacaagctttcccagaaaggatacagctggagtcagtttagtgatgtggaagagaacaggactgaggccccagaagggactgaatcg gagatggagacccccagtgccatcaatggcaacccatcctggcacctggcagacagccccgcggtgaatggagccactgGCcacagcagcagtttggatgcccg ggaggtgatccccatggcagcagtaaagcaagcgctgagggaggcaggcgacgagtttgaactgcggtaccggcgggcattcagtgacctgacatcccagctccac atcaccccagggacagcatatcagagctttgaacaggtagtgaatgaactcttccgggatggggtaaactggggtcgcattgtggcctttttctccttcggcggggcac tgtgcgtggaaagcgtagacaaggagatgcaggtattggtgagtcggatcgcagcttggatggccacttacctgaatgaccacctagagccttggatccaggagaac ggcggctgggatacttttgtggaactctatgggaacaatgcagcagccgagagccgaaagggccaggaacgcttcaaccgctggttcctgacgggcatgactgtgg ccggcgtggttctgctgggctcactcttCAGTCGGAAATGATCTAGACCTTCTGCGGGGCTTGCCTTCTGGCCATGCC CTTCTTCTCTCCCTTGCACCTGTACCTCTTGGTCTTTGAATAAAGCCTGAGTAGGAAAAAAAAAAAAA AAAAAAAAAAAAAAAAAAAAAAAAAAAAAAAAAAAAAAAAAAAAAAAAAAAAAAAAAAAAAAAAAAAAA AAAAAAAAAAAAAAAAAAAAAAAAAAAAAAAAAAAAA]

・1xMS2(U)site2-SpCas9

Template pDNA for PCR: pHL-EF1a-SphcCas9-iC-A

Primers for 1st PCR: MHC-1, MHC-2

Primers for 2nd PCR: HNC-242, HNC-396, HNC-440, 3'UTR oligo DNA

T7 promoter, MS2-binding motif (U), Kozak sequence (including start codon), SpCas9 gene

[CAGTGAATTGTAATACGACTCACTATAGGGCGAATTAAGAGAGAAAAGAAGAGTACATGAGGATTA CCCATGTAAGAAGAAATATAAGACACCGGTCGCCACCATGGATAAGAAATACAGCATTGGACTGGAC ATTGGGACAAACTCCGTGGGATGGGCCGTGATTACAGACGAATACAAAGTGCCTTCAAAGAAGTTCA AGGTGCTGGGCAACACCGATAGACACAGCATCAAGAAAAATCTGATTGGAGCCCTGCTGTTCGACTC CGGCGAGACAGCTGAAGCAACTCGGCTGAAAAGAACTGCTCGGAGAAGGTATACCCGCCGAAAGAA TAGGATCTGCTACCTGCAGGAGATTTTCAGCAACGAAATGGCCAAGGTGGACGATAGTTTCTTTCAC CGCCTGGAGGAATCATTCCTGGTCGAGGAAGATAAGAAACACGAGCGGCATCCCATCTTTGGCAACA TTGTGGACGAGGTCGCTTATCACGAAAAGTACCCTACCATCTATCATCTGAGGAAGAAACTGGTGGA CTCCACAGATAAAGCAGACCTGCGCCTGATCTATCTGGCCCTGGCTCACATGATTAAGTTCCGGGGC CATTTTCTGATCGAGGGGGATCTGAACCCAGACAATTCTGATGTGGACAAGCTGTTCATCCAGCTG GTCCAGACATACAATCAGCTGTTTGAGGAAAACCCCATTAATGCATCTGGCGTGGACGCAAAAGCCA TCCTGAGTGCCAGACTGTCTAAGAGTCGGAGACTGGAGAACCTGATCGCTCAGCTGCCAGGGGAAA AGAAAAACGGCCTGTTTGGGAATCTGATTGCACTGTCACTGGGACTGACTCCCAACTTCAAGAGCAA TTTTGATCTGGCCGAGGACGCTAAACTGCAGCTGTCCAAGGACACCTATGACGATGACCTGGATAAC CTGCTGGCTCAGATCGGGGATCAGTACGCAGACCTGTTCCTGGCCGCTAAGAATCTGTCTGACGCC ATCCTGCTGAGTGATATTCTGCGCGTGAACACCGAGATTACAAAAGCCCCCCTGTCAGCTAGCATGA TCAAGAGATATGACGAGCACCATCAGGATCTGACCCTGCTGAAGGCTCTGGTGAGGCAGCAGCTGC CTGAGAAGTACAAGGAAATCTTCTTTGATCAGTCTAAGAACGGATACGCCGGCTATATTGACGGCGG GGCTAGTCAGGAGGAGTTCTACAAGTTTATCAAACCCATTCTGGAGAAGATGGATGGCACAGAGGA ACTGCTGGTGAAACTGAATCGGGAAGACCTGCTGAGGAAGCAGCGCACTTTTGATAACGGAAGCAT CCCTCACCAGATTCATCTGGGAGAGCTGCACGCAATCCTGAGGCGCCAGGAAGACTTCTACCCATTT CTGAAGGATAACAGGGAGAAGATCGAAAAAATTCTGACATTCCGCATCCCCTACTATGTGGGCCCTC TGGCAAGAGGCAACAGCCGGTTTGCCTGGATGACTCGCAAATCTGAGGAAACAATCACTCCCTGGAA CTTCGAGGAAGTGGTCGATAAGGGCGCTTCCGCACAGTCTTTCATTGAGCGGATGACAAACTTCGA

CAAGAACCTGCCAAACGAAAAAGTGCTGCCCAAGCACTCTCTGCTGTACGAGTATTTCACAGTCTAT
AACGAACTGACTAAGGTGAAATACGTCACCGAGGGGATGAGAAAGCCTGCCTTCCTGAGTGGAGAA
CAGAAGAAAGCTATCGTGGACCTGCTGTTTAAAACCAATAGGAAGGTGACAGTCAAGCAGCTGAAAG
AGGACTATTTCAAGAAAATTGAATGTTTCGATTCTGTGGAGATCAGTGGCGTCGAAGACAGGTTTAA
CGCCTCCCTGGGGACCTACCACGATCTGCTGAAGATCATTAAGGATAAAGACTTCCTGGACAACGAG
GAAAATGAGGATATCCTGGAAGACATTGTGCTGACCCTGACACTGTTTGAGGATAGGGAAATGATC
GAGGAACGCCTGAAGACCTATGCCCATCTGTTCGATGACAAAGTGATGAAACAGCTGAAGCGACGGA
GATACACAGGATGGGGCCGACTGTCTCGGAAGCTGATCAATGGGATTCGCGACAAACAGAGTGGAA
AGACCATCCTGGACTTTCTGAAATCAGATGGCTTCGCCAACCGGAACTTCATGCAGCTGATTCACGA
TGACAGCCTGACATTCAAAGAGGATATCCAGAAGGCACAGGTGTCCGGGCAGGGAGACTCTCTGCA
CGAGCATATCGCAAACCTGGCCGGCAGCCCTGCCATCAAGAAAGGGATTCTGCAGACCGTGAAGGT
GGTGGACGAGCTGGTGAAAGTCATGGGAAGACATAAGCCAGAAAACATCGTGATTGAGATGGCCAG
GGAAAATCAGACCACACAGAAAGGCCAGAAGAACTCAAGGGAGCGCATGAAAAGAATCGAGGAAGGA
ATTAAGGAACTGGGCAGCCAGATCCTGAAAGAGCACCCCGTGGAAAACACACAGCTGCAGAATGAGA
AGCTGTATCTGTACTATCTGCAGAATGGACGCGATATGTACGTGGACCAGGAGCTGGATATTAACC
GACTGTCCGATTACGACGTGGATCATATCGTCCCACAGTCATTCCTGAAAGATGACAGCATTGACAA
TAAGGTGCTGACCCGCTCTGACAAAAACCGAGGCAAGAGTGATAATGTCCCCTCAGAGGAAGTGGT
CAAGAAAATGAAGAACTACTGGAGGCAGCTGCTGAATGCCAAACTGATCACACAGCGAAAGTTTGAT
AACCTGACTAAAGCTGAGCGGGGAGGCCTGAGTGAACTGGACAAAGCAGGCTTCATTAAGCGACAG
CTGGTGGAGACACGGCAGATCACAAAGCACGTCGCCCAGATTCTGGATTCAAGAATGAACACTAAGT
ACGATGAGAATGACAAACTGATCAGAGAAGTGAAGGTCATTACCCTGAAGTCAAAACTGGTGAGCGA
CTTTCGGAAAGATTTCCAGTTTTATAAGGTCAGAGAGATCAACAACTACCACCATGCTCATGACGCA
TACCTGAACGCAGTGGTCGGCACAGCCCTGATTAAGAAATACCCTAAACTGGAGTCCGAGTTCGTGT
ACGGGGACTATAAGGTGTACGATGTCAGAAAAATGATCGCCAAGTCTGAGCAGGAAATTGGCAAAG
CCACTGCTAAGTATTTCTTTTACAGTAACATCATGAATTTCTTTAAGACTGAGATCACCCTGGCAAAT
GGGGAAATCCGAAAGCGGCCACTGATTGAGACTAACGGCGAGACAGGAGAAATCGTGTGGGACAAA
GGAAGAGATTTTGCTACCGTGAGGAAGGTCCTGAGCATGCCCCAAGTGAATATTGTCAAGAAAACAG
AGGTGCAGACTGGGGGATTCAGTAAGGAATCAATTCTGCCTAAACGCAACTCCGATAAGCTGATCGC
CCGAAAGAAAGACTGGGACCCCAAGAAGTATGGCGGGTTCGACTCCCCAACTGTGGCTTACTCTGT
CCTGGTGGTCGCAAAGGTGGAGAAGGGAAAAAGCAAGAAACTGAAATCCGTCAAGGAACTGCTGGG
CATCACCATTATGGAGCGCAGCTCCTTCGAAAAGAATCCTATCGATTTTCTGGAGGCCAAAGGCTAT
AAGGAAGTGAAGAAAGACCTGATCATCAAGCTGCCAAAGTACTCACTGTTTGAGCTGGAAAACGGGA
GAAAGAGGATGCTGGCAAGCGCCGGGGAGCTGCAGAAAGGAAATGAACTGGCCCTGCCCTCCAAGT
ACGTGAACTTCCTGTATCTGGCTAGCCACTACGAGAAGCTGAAAGGGTCCCCTGAGGATAACGAACA
GAAACAGCTGTTTGTGGAGCAGCACAAGCATTATCTGGACGAGATCATTGAACAGATTAGCGAGTTC
TCCAAAAGAGTGATCCTGGCTGACGCAAATCTGGATAAGGTCCTGAGCGCATACAACAAACACCGGG
ATAAGCCAATCAGAGAGCAGGCCGAAAATATCATTCATCTGTTCACTCTGACCAACCTGGGAGCCCC
CGCAGCCTTCAAGTATTTTGACACTACCATCGATCGCAAACGATACACAAGCACTAAGGAGGTGCTG
GACGCTACCCTGATTCATCAGAGCATTACTGGCCTGTATGAAACAAGGATTGACCTGTCTCAGCTGG
GCGGCGACTCCGGAGCTGACCCCAAGAAGAAGAGGAAGGTGTGAATCTAGACCTTCTGCGGGGCTT
GCCTTCTGGCCATGCCCTTCTTCTCCCTTGCACCTGTACCTCTTGGTCTTTGAATAAAGCCTGAG
TAGGAAAAAAAAAAAAAAAAAAAAAAAAAAAAAAAAAAAAAAAAAAAAAAAAAAAAAAAAAA

AAAAAAAAAAAAAAAAAAAAAAAAAAAAAAAAAAAAAAAAAAAAAAAAAAAAAAAAAAAAAAAAA]

・2xScMS2(C)-AcrIIA4

Template pDNA for PCR: pHL-EF1a-AcrIIA4-iC-A

Primers for 1st PCR: MHC-144, MHC-145

Primers for 2nd PCR: HNC-370, HNC-396, 3'UTR oligo DNA

T7 promoter, 2xMS2-binding motif (C) with scaffold, Kozak sequence (including start codon), AcrIIA4 gene

[CAGTGAATTGTAATACGACTCACTATAGGGTCAGATCCGCTAGCGGATCCgggagcAggtgAGGATCACC
CATcTgccacgagcgAggtgAGGATCACCCATcTcgctcgtgttcccACCGGTCGCCACCATGAACATTAACGACCTC
ATACGAGAGATTAAGAACAAAGATTACACCGTCAAACTGTCAGGAACTGATAGTAACTCAATCACCCA
GCTTATTATCAGGGTAAACAATGATGGGAATGAATATGTGATATCTGAGAGCGAAAACGAGTCTATC
GTCGAGAAATTCATTTCCGCTTTTAAGAACGGGTGGAATCAGGAATATGAGGATGAAGAAGAATTTT
ACAATGACATGCAGACGATCACGTTGAAAAGTGAACTGAACTAAATCTAGACCTTCTGCGGGGCTTG
CCTTCTGGCCATGCCCTTCTTCTCTCCCTTGCACCTGTACCTCTTGGTCTTTGAATAAAGCCTGAGT
AGGAAAAAAAAAAAAAAAAAAAAAAAAAAAAAAAAAAAAAAAAAAAAAAAAAAAAAAAAAAAAAAAAAAAAAAAAAAAAAAA
AAAAAAAAAAAAAAAAAAAAAAAAAAAAAAAAAAAAAAAAAAAAAAAAAAAAAAAAAAAAAAAA]

・MS2CP-1xDmrA:

Template pDNA for PCR: pcDNA3.1-MS2CP-2xDmrA

Primers for 1st PCR: HNC-365, HNC-437

Primers for 2nd PCR: HNC-242, HNC-396, KEC-62, 3'UTR oligo DNA

T7 promoter, Kozak sequence (including start codon), MS2CP gene, DmrA gene

[CAGTGAATTGTAATACGACTCACTATAGGGCGAATTAAGAGAGAAAAGAAGAGTAAGAAGAAATATA
AGACACCGGTCGCCACCatgGCTTCTAACTTTACTCAGTTCGTTCTCGTCGACAATGGCGGAACTGGC
GACGTGACTGTCGCCCCAAGCAACTTCGCTAACGGGGTCGCTGAATGGATCAGCTCTAACTCGCGA
TCACAGGCTTACAAAGTAACCTGTAGCGTTCGTCAGAGCTCTGCGCAGAATCGCAAATACACCATCA
AAGTCGAGGTGCCTAAAGGCGCATGGAGGTCTTACTTAAATATGGAACTAACCATTCCAATTTTCGC
CACGAATTCCGACTGCGAGCTTATTGTTAAGGCAATGCAAGGTCTCCTAAAAGATGGAAACCCGATT
CCCTCGGCCATCGCGGCCAACTCCGGCATCTACGGATCCtctagaggagtgcaggtggaaaccatctccccaggagacgggcg
caccttccccaagcgcggccagacctgcgtggtgcactacaccgggatgcttgaagatggaaagaaatttgattcctcccgggacagaaacaagccctttaagtttat
gctaggcaagcaggaggtgatccgaggctgggaagaaggggttgcccagatgagtgtgggtcagagagccaaactgactatatctccagattatgcctatggtgcca
ctgggcacccaggcatcatcccaccacatgccactctcgtcttcgatgtggagcttctaaaactggaaTGAATCTAGACCTTCTGCGGGGCTT
GCCTTCTGGCCATGCCCTTCTTCTCTCCCTTGCACCTGTACCTCTTGGTCTTTGAATAAAGCCTGAG
TAGGAAAAAAAAAAAAAAAAAAAAAAAAAAAAAAAAAAAAAAAAAAAAAAAAAAAAAAAAAAAAAAAAAAAAAAAAAAAA
AAAAAAAAAAAAAAAAAAAAAAAAAAAAAAAAAAAAAAAAAAAAAAAAAAAAAAAAAAAAAAAAA]

・MS2CP(V29I)-1xDmrA:

Template pDNA for PCR: pcDNA3.1-MS2CP(V29I)-2xDmrA

Primers for 1st PCR: HNC-365, HNC-437

Primers for 2nd PCR: HNC-242, HNC-396, KEC-62, 3'UTR oligo DNA

T7 promoter, Kozak sequence (including start codon), MS2CP(V29I) gene, DmrA gene

[CAGTGAATTGTAATACGACTCACTATAGGGCGAATTAAGAGAGAAAAGAAGAGTAAGAAGAAATATA

AGACACCGGTCGCCACCatgGCTTCTAACTTTACTCAGTTCGTTCTCGTCGACAATGGCGGAACTGGC
GACGTGACTGTCGCCCCAAGCAACTTCGCTAACGGGATCGCTGAATGGATCAGCTCTAACTCGCGAT
CACAGGCTTACAAAGTAACCTGTAGCGTTCGTCAGAGCTCTGCGCAGAATCGCAAATACACCATCAA
AGTCGAGGTGCCTAAAGGCGCATGGAGGTCTTACTTAAATATGGAACTAACCATTCCAATTTTCGCC
ACGAATTCCGACTGCGAGCTTATTGTTAAGGCAATGCAAGGTCTCCTAAAAGATGGAAACCCGATTC
CCTCGGCCATCGCGGCCAACTCCGGCATCTACGGATCCtctagaggagtgcaggtggaaaccatctccccaggagacgggcgc
accttccccaagcgcggccagacctgcgtggtgcactacaccgggatgcttgaagatggaaagaaatttgattcctcccgggacagaaacaagccctttaagtttatg
ctaggcaagcaggaggtgatccgaggctgggaagaaggggttgcccagatgagtgtgggtcagagagccaaactgactatatctccagattatgcctatggtgccac
tgggcacccaggcatcatcccaccacatgccactctcgtcttcgatgtggagcttctaaaactggaaTGAATCTAGACCTTCTGCGGGGCTT
GCCTTCTGGCCATGCCCTTCTTCTCTCCCTTGCACCTGTACCTCTTGGTCTTTGAATAAAGCCTGAG
TAGGAAAAAAAAAAAAAAAAAAAAAAAAAAAAAAAAAAAAAAAAAAAAAAAAAAAAAAAAAAAAAAA
AAAAAAAAAAAAAAAAAAAAAAAAAAAAAAAAAAAAAAAAAAAAAAAAAAAAA]

・DmrC-VPg(FCV)

Template pDNA for PCR: pcDNA3.1-DmrC-VPg(FCV)

Primers for 1st PCR: HNC-395, HNC-266

Primers for 2nd PCR: HNC-242, HNC-396, KEC-62, 3'UTR oligo DNA

T7 promoter, Kozak sequence (including start codon), DmrC gene, VPg(FCV) gene, DYK-tag gene

[CAGTGAATTGTAATACGACTCACTATAGGGCGAATTAAGAGAGAAAAGAAGAGTAAGAAGAAATATA
AGACACCGGTCGCCACCatggcttctagaatcctctggcatgagatgtggcatgaaggcctggaagaggcatctcgtttgtactttggggaaaggaac
gtgaaaggcatgtttgaggtgctggagcccttgcatgctatgatggaacggggcccccagactctgaaggaaacatcctttaatcaggcctatggtcgagatttaatgg
aggcccaagagtggtgcaggaagtacatgaaatcagggaatgtcaaggacctcctccaagcctgggacctctattatcatgtgttccgacgaatctcaaagGGAT
CCGCCAAGGGCAAGACCAAGAGCAAAGTGGGCCCCTACAGAGGCAGAGGCGTGGCCCTTACAGACG
ACGAGTATGACGAATGGCGCGAGCACAACGCCACCAGAAAGCTGGATCTGAGCGTGGAAGATTTCC
TGATGCTGCGGCACAGAGCCGCTCTGGGAGCTGATGATGCCGACGCCGTGAAGTTCAGATCCTGGT
GGAACAGCAGAAGCCGGCTGGCCGACGATTACGAGGATGTGACCGTGATCGGCAAAGGCGGCGTGA
AGCACGAGAAGATCCGGACCAATACTCTGAGAGCCGTGGACAGAGGCTACGACGTGTCCTTCGCTG
AAGAAACCGGTGTTCCAACTGTTGATTACAAGGATGACGACGATAAGTGAATCTAGACCTTCTGCGG
GGCTTGCCTTCTGGCCATGCCCTTCTTCTCTCCCTTGCACCTGTACCTCTTGGTCTTTGAATAAAGC
CTGAGTAGGAAAAAAAAAAAAAAAAAAAAAAAAAAAAAAAAAAAAAAAAAAAAAAAAAAAAAAAAAA
AAAAAAAAAAAAAAAAAAAAAAAAAAAAAAAAAAAAAAAAAAAAAAAAAAAAAAAAAAA]

・EGFP-targeting sgRNA

Primers for PCR: U15K160429, MHC-4

T7 promoter, EGFP-targeting region

[GCTAATACGACTCACTATAgggcacgggcagcttgccggGTTTTAGAGCTAGAAATAGCAagttaaaataaggctagtccgtt
atcaacttgaaaaagtggcaccgagtcggtgcttt]

List of primers to prepare templates for in vitro transcription

HNC-237	CACCGGTCGCCACCATGgtgtctaagggcgaagagctga
HNC-238	GCCCCGCAGAAGGTCTAGATTCAattaagtttgtgccccagtttg
HNC-242	CAGTGAATTGTAATACGACTCACTATAGGGCGA
HNC-266	GCCCCGCAGAAGGTCTAGATTCACTTATCGTCGTCATCCTTG
HNC-365	CACCGGTCGCCACCATGGCTTCTAACTTTAC
HNC-370	CAGTGAATTGTAATACGACTCACTATAGGGTCAGATCCGCTAGCGGATCCgggagcAggtgAGGATCACCCATcTgccacgagcgAggtgAGGATCACCCATcTcgctcgtgttcccACCGGTCGCCACCATG
HNC-372	CACCGGTCGCCACCATGGCCAAGGGCAAGACCAAGAGCA
HNC-373	CACCGGTCGCCACCATGGGCAAGAACAAGGGCAAGACCA
HNC-378	CAGTGAATTGTAATACGACTCACTATAGGGCGAATTAAGAGAGAAAAGAAGAGTAAGAAGAAATATAAGACACC
HNC-379	GCCCCGCAGAAGGTCTAGATTCAGTAGATGCCGGAGTTGG
HNC-382	CAGTGAATTGTAATACGACTCACTATAGGGACATGAGGATCACCCATGTCGAATTAAGAGAGAAAAGAAGAGTAAGAAGAAATATAAGACACC
HNC-383	AGAAAAGAAGAGTAAGAAGAAATATAAGACACCGGTCGCCACCATGGTGAGCGTGATCAAGCCCGAGA
HNC-395	CACCGGTCGCCACCATGGCTTCTAGAATC
HNC-396	TTTTTTTTTTTTTTTTTTTTTTTTTTTTTTTTTTTTTTTTTTTTTTTTTTTTTTTTTTTTTTTTTTTTTTTTTTTTTTTTTTTTTTTTTTTTTTTTTTTTTTTTTTTTTTTTTTTTTTTTCCTACTCAGGCTTTATTCA
HNC-401	AGAAAAGAAGAGTAAGAAGAAATATAAGACACCGGTCTCAACATCAGTCTGATAAGCTAGCCACCATGGCTTCTAACTTTAC
HNC-408	CAGTGAATTGTAATACGACTCACTATAGGGACATGAGGATTACCCATGTCGAATTAAGAGAGAAAAGAAGAGTAAGAAGAAATATAAGACACC
HNC-437	GCCCCGCAGAAGGTCTAGATTCATTCCAGTTTTAGAAGCTCCACATCG
HNC-440	TGTAATACGACTCACTATAGGGCGAATTAAGAGAGAAAAGAAGAGTACATGAGGATTACCCATGTAAGAAGAAATATAAGACACCGGTCGCCACCATG
HNC-441	AGAAAAGAAGAGTAAGAAGAAATATAAGACACCGGTCACATGAGGATTACCCATGTGCCACCATGGTGAGCGTGATCAAGCCCGAGA
HNC-446	TGTAATACGACTCACTATAGGGCGAATTAAGAGAGAAAAGAAGAGTACATGAGGATCACCCATGTAAGAAGAAATATAAGACACCGGTCGCCACCATG
HNC-447	AGAAAAGAAGAGTAAGAAGAAATATAAGACACCGGTCACATGAGGATCACCCATGTGCCACCATGGTGAGCGTGATCAAGCCCGAGA
HNC-475	AGAAAAGAAGAGTAAGAAGAAATATAAGACACCGGTCAGCAAGTACATCCACGTTTAAGTGCCACCATGGCTTCTAACTTTAC
KEC-62	CAGTGAATTGTAATACGACTCACTATAGGGCGAATTAAGAGAGAAAAGAAGAGTAAGAAGAAATATAAGACACCGGTCGCCACCATG
KEC-330	CACCGGTCGCCACCATGGTGAGCGTGATCAAGCCCG
KEC-331	GCCCCGCAGAAGGTCTAGATTCACTTGGCCTGGCTGGGC
MHC-1	CACCGGTCGCCACCATGGATAAGAAATACAGCATTGGAC

MHC-2	GCCCCGCAGAAGGTCTAGACTATCACACCTTCCTCTTCTTCTTGG
MHC-4	aaagcaccgactcggtgccactttttcaagttgataacggactagccttattttaactTGCTATTTCTAGCTCTAAAAC
MHC-144	CACCGGTCGCCACCATGAACATTAACGACCTCATACGAG
MHC-145	GCCCCGCAGAAGGTCTAGACTATTAGTTCAGTTCACTTTTCAACGTG
U15K160429	GCTAATACGACTCACTATAGGGCACGGGCAGCTTGCCGGGTTTTAGAGCTAGAAATAGCAAG
3’UTR oligo DNA (double strand)	TCTAGACCTTCTGCGGGGCTTGCCTTCTGGCCATGCCCTTCTTCTCTCCCTTGCACCTGTACCTCTTGGTCTTTGAATAAAGCCTGAGTAGGAAAAAAAAAAAAAAAAAAAAA

List of transfection conditions

Figure 2, Supplementary Figure 1

<table>
<tr><th>Transfection control mRNA
・ cap analog: ARCA
・ modified nucleosides: N1mΨ
・ 100 ng/well</th><th>Target mRNA
・ cap analog: A-cap
・ modified nucleosides: N1mΨ
・ 360 ng/well</th><th>Translational effector mRNA
・ cap analog: ARCA
・ modified nucleosides: N1mΨ
・ 40 ng/well</th></tr>
<tr><td rowspan="12">tagRFP</td><td rowspan="6">hmAG1</td><td>-</td></tr>
<tr><td>MS2CP</td></tr>
<tr><td>MS2CP-VPg(NV-GI)</td></tr>
<tr><td>CaVT</td></tr>
<tr><td>VPg(NV-GI)</td></tr>
<tr><td>VPg(FCV)</td></tr>
<tr><td rowspan="6">1xMS2(C)site1-hmAG1</td><td>-</td></tr>
<tr><td>MS2CP</td></tr>
<tr><td>MS2CP-VPg(NV-GI)</td></tr>
<tr><td>CaVT</td></tr>
<tr><td>VPg(NV-GI)</td></tr>
<tr><td>VPg(FCV)</td></tr>
</table>

Supplementary Figure 2

<table>
<tr><th>Transfection control mRNA
・ cap analog: ARCA
・ modified nucleosides: N1mΨ
・ 100 ng/well</th><th>Target mRNA
・ cap analog: ARCA
・ modified nucleosides: N1mΨ
・ 50 ng/well</th><th>Translational effector mRNA
・ cap analog: ARCA
・ modified nucleosides: N1mΨ
・ 40 ng/well</th></tr>
<tr><td rowspan="2">tagRFP</td><td rowspan="2">1xMS2(C)site1-hmAG1</td><td>-</td></tr>
<tr><td>CaVT</td></tr>
</table>

Figure 3, Supplementary Figure 3

Transfection control mRNA<br>・ cap analog: ARCA<br>・ modified nucleosides: N1mΨ<br>・ 100 ng/well	Target mRNA<br>・ cap analog: A-cap<br>・ 360 ng/well	Translational effector mRNA<br>・ cap analog: ARCA<br>・ modified nucleosides: N1mΨ<br>・ 40 ng/well
tagRFP	1xMS2(C)site1-hmAG1 (modified nucleosides: N1mΨ)	-
		CaVT
		CaVT (V29I mutant)
	1xMS2(C)site2-hmAG1 (modified nucleosides: N1mΨ)	-
		CaVT
		CaVT (V29I mutant)
	1xMS2(C)site3-hmAG1 (modified nucleosides: N1mΨ)	-
		CaVT
		CaVT (V29I mutant)
	1xMS2(U)site1-hmAG1 (modified nucleosides: N1mΨ)	-
		CaVT
		CaVT (V29I mutant)
	1xMS2(U)site2-hmAG1 (modified nucleosides: N1mΨ)	-
		CaVT
		CaVT (V29I mutant)
	1xMS2(U)site3-hmAG1 (modified nucleosides: N1mΨ)	-
		CaVT
		CaVT (V29I mutant)
	1xMS2(C)site1-hmAG1 (modified nucleosides: Ψ/5mC)	-
		CaVT
		CaVT (V29I mutant)
	1xMS2(C)site2-hmAG1 (modified nucleosides: Ψ/5mC)	-
		CaVT
		CaVT (V29I mutant)
	1xMS2(C)site3-hmAG1 (modified nucleosides: Ψ/5mC)	-
		CaVT
		CaVT (V29I mutant)
	1xMS2(U)site1-hmAG1 (modified nucleosides: Ψ/5mC)	-
		CaVT
		CaVT (V29I mutant)
	1xMS2(U)site2-hmAG1 (modified nucleosides: Ψ/5mC)	-
		CaVT
		CaVT (V29I mutant)
	1xMS2(U)site3-hmAG1 (modified nucleosides: Ψ/5mC)	-
		CaVT
		CaVT (V29I mutant)

Supplementary Figure 4, 5

Transfection control mRNA<br>・ cap analog: ARCA<br>・ modified nucleosides: N1mΨ<br>・ 100 ng/well	Target mRNA<br>・ cap analog: A-cap<br>・ 320 ng/well	Translational effector mRNA<br>・ cap analog: ARCA<br>・ modified nucleosides: N1mΨ<br>・ 80 ng/well
tagRFP	1xMS2(C)site1-hmAG1<br>(modified nucleosides: N1mΨ)	-
		CaVT
		CaVT (V29I mutant)
	1xMS2(C)site2-hmAG1<br>(modified nucleosides: N1mΨ)	-
		CaVT
		CaVT (V29I mutant)
	1xMS2(C)site3-hmAG1<br>(modified nucleosides: N1mΨ)	-
		CaVT
		CaVT (V29I mutant)
	1xMS2(U)site1-hmAG1<br>(modified nucleosides: N1mΨ)	-
		CaVT
		CaVT (V29I mutant)
	1xMS2(U)site2-hmAG1<br>(modified nucleosides: N1mΨ)	-
		CaVT
		CaVT (V29I mutant)
	1xMS2(U)site3-hmAG1<br>(modified nucleosides: N1mΨ)	-
		CaVT
		CaVT (V29I mutant)
	1xMS2(C)site1-hmAG1<br>(modified nucleosides: Ψ/5mC)	-
		CaVT
		CaVT (V29I mutant)
	1xMS2(C)site2-hmAG1<br>(modified nucleosides: Ψ/5mC)	-
		CaVT
		CaVT (V29I mutant)
	1xMS2(C)site3-hmAG1<br>(modified nucleosides: Ψ/5mC)	-
		CaVT
		CaVT (V29I mutant)
	1xMS2(U)site1-hmAG1<br>(modified nucleosides: Ψ/5mC)	-
		CaVT
		CaVT (V29I mutant)
	1xMS2(U)site2-hmAG1<br>(modified nucleosides: Ψ/5mC)	-
		CaVT
		CaVT (V29I mutant)
	1xMS2(U)site3-hmAG1<br>(modified nucleosides: Ψ/5mC)	-
		CaVT
		CaVT (V29I mutant)

Figure 4b-d, Supplementary Figure 6a

Transfection control mRNA<br>・ cap analog: ARCA<br>・ modified nucleosides: N1mΨ<br>・ 100 ng/well	Target mRNA<br>・ cap analog: A-cap<br>・ modified nucleosides: N1mΨ<br>・ 360 ng/well	Translational effector mRNA<br>・ cap analog: ARCA<br>・ modified nucleosides: N1mΨ<br>・ 40 ng/well
tagRFP	2xScMS2(C)-hmAG1	-
		CaVT
		CaVT (V29I mutant)

Figure 4e, Supplementary Figure 6b

Transfection control mRNA<br>・ cap analog: ARCA<br>・ modified nucleosides: N1mΨ<br>・ 100 ng/well	Target mRNA<br>・ cap analog: ARCA<br>・ modified nucleosides: N1mΨ<br>・ 20 ng/well	Translational effector mRNA<br>・ cap analog: ARCA<br>・ modified nucleosides: N1mΨ<br>・ 40 ng/well
tagRFP	2xScMS2(C)-hmAG1	-
		CaVT
		CaVT (V29I mutant)

Supplementary Figure 7

Transfection control mRNA<br>・ cap analog: ARCA<br>・ modified nucleosides: N1mΨ<br>・ 100 ng/well	Target mRNA<br>・ cap analog: A-cap<br>・ modified nucleosides: N1mΨ<br>・ 320 ng/well	Translational effector mRNA<br>・ cap analog: ARCA<br>・ modified nucleosides: N1mΨ<br>・ 80 ng/well
tagRFP	2xScMS2(C)-hmAG1	-
		CaVT
		CaVT (V29I mutant)

Figure 5b

Pro-apoptotic mRNA<br>・ cap analog: A-cap<br>・ modified nucleosides: N1mΨ<br>・ 90 ng/well	Anti-apoptotic mRNA<br>・ cap analog: ARCA<br>・ modified nucleosides: N1mΨ<br>・ 20 ng/well	Translational effector mRNA<br>・ cap analog: ARCA<br>・ modified nucleosides: N1mΨ
-	-	-
		CaVT (15 ng/well)
		CaVT (30 ng/well)
		MS2CP (30 ng/well)
1xMS2(U)site2-Bax	-	-
		CaVT (15 ng/well)
		CaVT (30 ng/well)
		MS2CP (30 ng/well)
	2xScMS2(C)-BclxL	-
		CaVT (15 ng/well)
		CaVT (30 ng/well)
		MS2CP (30 ng/well)

Figure 5c, d

Pro-apoptotic mRNA<br>・ modified nucleosides: N1mΨ<br>・ 360 ng/well	Anti-apoptotic mRNA<br>・ cap analog: ARCA<br>・ modified nucleosides: N1mΨ<br>・ 80 ng/well	Translational effector mRNA<br>・ cap analog: ARCA<br>・ modified nucleosides: N1mΨ<br>・ 120 ng/well
-	-	-
1xMS2(U)site2-Bax<br>(cap analog: ARCA)	-	-
1xMS2(U)site2-Bax<br>(cap analog: A-cap)	-	-
		CaVT
	2xScMS2(C)-BclxL	-
		CaVT

Figure 6

<table>
<tr><th>Cas9 mRNA
・ modified nucleosides: N1mΨ
・ 360 ng/well</th><th>sgRNA
・ 25 ng/well</th><th>AcrIIA4 mRNA
・ cap analog: ARCA
・ modified nucleosides: N1mΨ</th><th>Translational effector mRNA
・ cap analog: ARCA
・ modified nucleosides: N1mΨ
・ 120 ng/well</th></tr>
<tr><td>-</td><td>-</td><td>-</td><td>-</td></tr>
<tr><td rowspan="2">1xMS2(U)site2-SpCas9
(cap analog: ARCA)</td><td>-</td><td>-</td><td>-</td></tr>
<tr><td>EGFP-targeting</td><td>-</td><td>-</td></tr>
<tr><td rowspan="6">1xMS2(U)site2-SpCas9
(cap analog: A-cap)</td><td rowspan="6">EGFP-targeting</td><td rowspan="2">-</td><td>-</td></tr>
<tr><td>CaVT</td></tr>
<tr><td rowspan="2">2xScMS2(C)-AcrIIA4
(0.5 ng/well)</td><td>-</td></tr>
<tr><td>CaVT</td></tr>
<tr><td rowspan="2">2xScMS2(C)-AcrIIA4
(1 ng/well)</td><td>-</td></tr>
<tr><td>CaVT</td></tr>
</table>

Supplementary Figure 8

<table>
<tr><th>Pro-apoptotic mRNA
・ cap analog: A-cap
・ modified nucleosides: N1mΨ
・ 90 ng/well</th><th>Anti-apoptotic mRNA
・ cap analog: ARCA
・ modified nucleosides: N1mΨ
・ 20 ng/well</th><th>Translational effector mRNA
・ cap analog: ARCA
・ modified nucleosides: N1mΨ
・ 30 ng/well</th><th>miRNA mimic or inhibitor
・ 0.3 pmol/well</th></tr>
<tr><td>-</td><td>-</td><td>-</td><td>-</td></tr>
<tr><td rowspan="8">1xMS2(U)site2-Bax</td><td rowspan="8">2xScMS2(C)-BclxL</td><td rowspan="2">CaVT</td><td>miR-302a-5p mimic</td></tr>
<tr><td>Control mimic</td></tr>
<tr><td rowspan="2">miR-302a-5p-responsive CaVT</td><td>miR-302a-5p mimic</td></tr>
<tr><td>Control mimic</td></tr>
<tr><td rowspan="2">CaVT</td><td>Control inhibitor</td></tr>
<tr><td>miR-21-5p inhibitor</td></tr>
<tr><td rowspan="2">miR-21-5p-responsive CaVT</td><td>Control inhibitor</td></tr>
<tr><td>miR-21-5p inhibitor</td></tr>
</table>

Figure 7b, Supplementary Figure 9

<table>
<tr><th>Pro-apoptotic mRNA
・ modified nucleosides: N1mΨ
・ 360 ng/well</th><th>Anti-apoptotic mRNA
・ cap analog: ARCA
・ modified nucleosides: N1mΨ
・ 80 ng/well</th><th>Translational effector mRNA
・ cap analog: ARCA
・ modified nucleosides: N1mΨ
・ 120 ng/well</th><th>miRNA mimic or inhibitor
・ 1.2 pmol/well</th></tr>
<tr><td>-</td><td>-</td><td>-</td><td>-</td></tr>
<tr><td>1xMS2(U)site2-Bax
(cap analog: ARCA)</td><td>-</td><td>-</td><td>-</td></tr>
<tr><td rowspan="8">1xMS2(U)site2-Bax
(cap analog: A-cap)</td><td rowspan="8">2xScMS2(C)-BclxL</td><td rowspan="2">miR-302a-5p-responsive CaVT</td><td>miR-302a-5p mimic</td></tr>
<tr><td>Control mimic</td></tr>
<tr><td rowspan="2">CaVT</td><td>miR-302a-5p mimic</td></tr>
<tr><td>Control mimic</td></tr>
<tr><td rowspan="2">miR-21-5p-responsive CaVT</td><td>miR-21-5p inhibitor</td></tr>
<tr><td>Control inhibitor</td></tr>
<tr><td rowspan="2">CaVT</td><td>miR-21-5p inhibitor</td></tr>
<tr><td>Control inhibitor</td></tr>
</table>

Figure 7d, Supplementary Figure 10

<table>
<tr><th>Cas9 mRNA
・ modified nucleosides: N1mΨ
・ 360 ng/well</th><th>sgRNA
・ 25 ng/well</th><th>AcrIIA4 mRNA
・ cap analog: ARCA
・ modified nucleosides: N1mΨ
・ 0.5 ng/well</th><th>Translational effector mRNA
・ cap analog: ARCA
・ modified nucleosides: N1mΨ
・ 60 ng/well</th></tr>
<tr><td>-</td><td>-</td><td>-</td><td>-</td></tr>
<tr><td rowspan="2">1xMS2(U)site2-SpCas9
(cap analog: ARCA)</td><td>-</td><td>-</td><td>-</td></tr>
<tr><td>EGFP-targeting</td><td>-</td><td>-</td></tr>
<tr><td rowspan="4">1xMS2(U)site2-SpCas9
(cap analog: A-cap)</td><td rowspan="4">EGFP-targeting</td><td rowspan="4">2xScMS2(C)-AcrIIA4</td><td>-</td></tr>
<tr><td>miR-302a-5p-responsive CaVT</td></tr>
<tr><td>miR-21-5p-responsive CaVT</td></tr>
<tr><td>CaVT</td></tr>
</table>

Figure 8b, c, Supplementary Figure 11, 12

Transfection control mRNA<br>・ cap analog: ARCA<br>・ modified nucleosides: N1mΨ<br>・ 100 ng/well	Target mRNA<br>・ cap analog: A-cap<br>・ 320 ng/well	Translational effector mRNA<br>・ cap analog: ARCA<br>・ modified nucleosides: N1mΨ
tagRFP	1xMS2(C)site1-hmAG1<br>(modified nucleosides: N1mΨ)	-
		MS2CP-1xDmrA (20 ng/well)<br>+ DmrC-VPg (60 ng/well)
		MS2CP(V29I)-1xDmrA (20 ng/well)<br>+ DmrC-VPg (60 ng/well)
	1xMS2(C)site2-hmAG1<br>(modified nucleosides: N1mΨ)	-
		MS2CP-1xDmrA (20 ng/well)<br>+ DmrC-VPg (60 ng/well)
		MS2CP(V29I)-1xDmrA (20 ng/well)<br>+ DmrC-VPg (60 ng/well)
	1xMS2(C)site3-hmAG1<br>(modified nucleosides: N1mΨ)	-
		MS2CP-1xDmrA (20 ng/well)<br>+ DmrC-VPg (60 ng/well)
		MS2CP(V29I)-1xDmrA (20 ng/well)<br>+ DmrC-VPg (60 ng/well)
	1xMS2(U)site1-hmAG1<br>(modified nucleosides: N1mΨ)	-
		MS2CP-1xDmrA (20 ng/well)<br>+ DmrC-VPg (60 ng/well)
		MS2CP(V29I)-1xDmrA (20 ng/well)<br>+ DmrC-VPg (60 ng/well)
	1xMS2(U)site2-hmAG1<br>(modified nucleosides: N1mΨ)	-
		MS2CP-1xDmrA (20 ng/well)<br>+ DmrC-VPg (60 ng/well)
		MS2CP(V29I)-1xDmrA (20 ng/well)<br>+ DmrC-VPg (60 ng/well)
	1xMS2(U)site3-hmAG1<br>(modified nucleosides: N1mΨ)	-
		MS2CP-1xDmrA (20 ng/well)<br>+ DmrC-VPg (60 ng/well)
		MS2CP(V29I)-1xDmrA (20 ng/well)<br>+ DmrC-VPg (60 ng/well)
	1xMS2(C)site1-hmAG1<br>(modified nucleosides: Ψ/5mC)	-
		MS2CP-1xDmrA (20 ng/well)<br>+ DmrC-VPg (60 ng/well)
		MS2CP(V29I)-1xDmrA (20 ng/well)<br>+ DmrC-VPg (60 ng/well)
	1xMS2(C)site2-hmAG1<br>(modified nucleosides: Ψ/5mC)	-
		MS2CP-1xDmrA (20 ng/well)

		+ DmrC-VPg (60 ng/well)
		MS2CP(V29I)-1xDmrA (20 ng/well) + DmrC-VPg (60 ng/well)
1xMS2(C)site3-hmAG1 (modified nucleosides: Ψ/5mC)		-
		MS2CP-1xDmrA (20 ng/well) + DmrC-VPg (60 ng/well)
		MS2CP(V29I)-1xDmrA (20 ng/well) + DmrC-VPg (60 ng/well)
	1xMS2(U)site1-hmAG1 (modified nucleosides: Ψ/5mC)	-
		MS2CP-1xDmrA (20 ng/well) + DmrC-VPg (60 ng/well)
		MS2CP(V29I)-1xDmrA (20 ng/well) + DmrC-VPg (60 ng/well)
	1xMS2(U)site2-hmAG1 (modified nucleosides: Ψ/5mC)	-
		MS2CP-1xDmrA (20 ng/well) + DmrC-VPg (60 ng/well)
		MS2CP(V29I)-1xDmrA (20 ng/well) + DmrC-VPg (60 ng/well)
	1xMS2(U)site3-hmAG1 (modified nucleosides: Ψ/5mC)	-
		MS2CP-1xDmrA (20 ng/well) + DmrC-VPg (60 ng/well)
		MS2CP(V29I)-1xDmrA (20 ng/well) + DmrC-VPg (60 ng/well)

Supplementary Figure 13

Transfection control mRNA<br>・ cap analog: ARCA<br>・ modified nucleosides: N1mΨ<br>・ 100 ng/well	Target mRNA<br>・ cap analog: A-cap<br>・ modified nucleosides: N1mΨ<br>・ 320 ng/well	Translational effector mRNA<br>・ cap analog: ARCA<br>・ modified nucleosides: N1mΨ
tagRFP	1xMS2(U)site1-hmAG1	MS2CP-1xDmrA (20 ng/well) + DmrC-VPg (60 ng/well)
		MS2CP-1xDmrA (20 ng/well)
		DmrC-VPg (60 ng/well)
		-

Figure 8d, e, Supplementary Figure 14

Transfection control mRNA<br>・ cap analog: ARCA<br>・ modified nucleosides: N1mΨ<br>・ 100 ng/well	Target mRNA<br>・ cap analog: A-cap<br>・ modified nucleosides: N1mΨ<br>・ 320 ng/well	Translational effector mRNA<br>・ cap analog: ARCA<br>・ modified nucleosides: N1mΨ
tagRFP	1xMS2(U)site1-hmAG1	MS2CP-1xDmrA (20 ng/well) + DmrC-VPg (60 ng/well)

Figure 8f, Supplementary Video 1, 2

Target mRNA<br>・ cap analog: A-cap<br>・ modified nucleosides: N1mΨ<br>・ 420 ng/well	Translational effector mRNA<br>・ cap analog: ARCA<br>・ modified nucleosides: N1mΨ
1xMS2(U)site1-hmAG1	MS2CP-1xDmrA (20 ng/well)<br>+ DmrC-VPg (60 ng/well)

Gating strategy

1. hmAG1/tagRFP reporter assay

Cells inside forward and side scatter gates (an example is shown below) were used to visualize hmAG1-tagRFP dot plots.

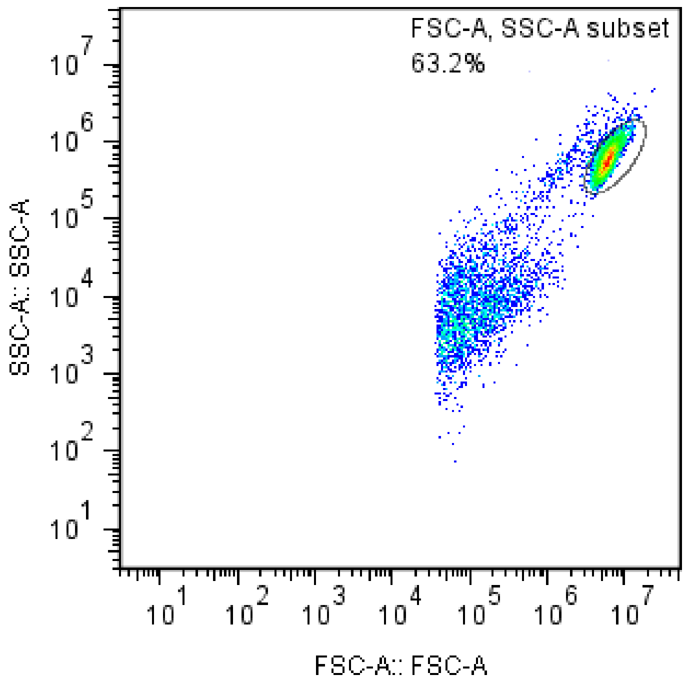

Cross gates were set based on hmAG1-tagRFP dot plots of untransfected samples (an example is shown below). In the visualization and calculation of the hmAG1/tagRFP ratio, hmAG1(+)-tagRFP(+) regions (Q6 region in the example) were used to reduce the influence of autofluorescence.

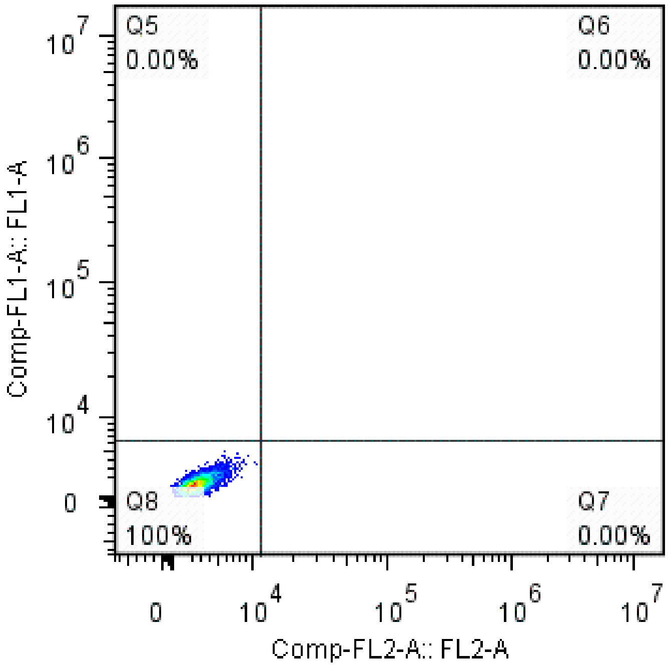

2. Apoptosis assay

Cells inside forward and side scatter gates (an example is shown below) were used to visualize Annexin V-SYTOX Red dot plots. Different from the case of the hmAG1/tagRFP reporter assay, the gates were set to include both live and dead cells.

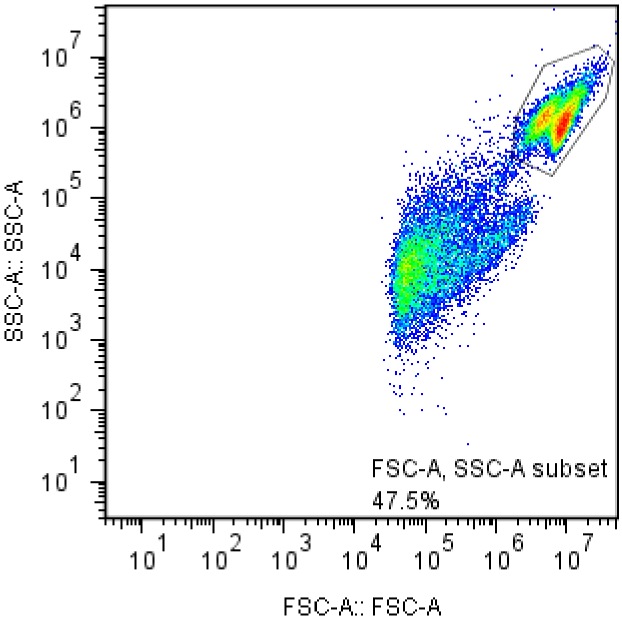

3. EGFP knockout assay

A forward and side scatter gating strategy similar to the case of hmAG1/tagRFP reporter assay was used.

Supplementary References

1. Nakanishi, H. *et al.* Monitoring and visualizing microRNA dynamics during live cell differentiation using microRNA-responsive non-viral reporter vectors. *Biomaterials* **128**, 121–135 (2017).
