# Supplementary material for: Caliciviral protein-based artificial translational activator for mammalian gene circuits with RNA-only delivery
Source: Nat Commun. 2020 Mar 10;11:1297. doi: 10.1038/s41467-020-15061-x (PMC7064597; doi:10.1038/s41467-020-15061-x)
Supplement: Supplementary file 3 — Reporting Summary [file 41467_2020_15061_MOESM3_ESM.pdf]

nature research

Corresponding author(s): Hirohide Saito

Last updated by author(s): Feb 1, 2020

Reporting Summary

Nature Research wishes to improve the reproducibility of the work that we publish. This form provides structure for consistency and transparency in reporting. For further information on Nature Research policies, see Authors & Referees and the Editorial Policy Checklist .

Statistics

For all statistical analyses, confirm that the following items are present in the figure legend, table legend, main text, or Methods section.

n/a	Confirmed	
☐	☒	The exact sample size (n) for each experimental group/condition, given as a discrete number and unit of measurement
☐	☒	A statement on whether measurements were taken from distinct samples or whether the same sample was measured repeatedly
☐	☒	The statistical test(s) used AND whether they are one- or two-sided<br>*Only common tests should be described solely by name; describe more complex techniques in the Methods section.*
☒	☐	A description of all covariates tested
☒	☐	A description of any assumptions or corrections, such as tests of normality and adjustment for multiple comparisons
☐	☒	A full description of the statistical parameters including central tendency (e.g. means) or other basic estimates (e.g. regression coefficient) AND variation (e.g. standard deviation) or associated estimates of uncertainty (e.g. confidence intervals)
☐	☒	For null hypothesis testing, the test statistic (e.g. F, t, r) with confidence intervals, effect sizes, degrees of freedom and P value noted<br>*Give P values as exact values whenever suitable.*
☒	☐	For Bayesian analysis, information on the choice of priors and Markov chain Monte Carlo settings
☒	☐	For hierarchical and complex designs, identification of the appropriate level for tests and full reporting of outcomes
☒	☐	Estimates of effect sizes (e.g. Cohen's d, Pearson's r), indicating how they were calculated

Our web collection on statistics for biologists contains articles on many of the points above.

Software and code

Policy information about availability of computer code

Data collection	CFlow Plus (version 1.0.227.4)
Data analysis	FlowJo7.6.5, R x64 3.6.1, Microsoft Excel for Office 365 (version 1902), Jupyter Notebook 6.0.1, ImageJ 1.51j8

For manuscripts utilizing custom algorithms or software that are central to the research but not yet described in published literature, software must be made available to editors/reviewers. We strongly encourage code deposition in a community repository (e.g. GitHub). See the Nature Research guidelines for submitting code & software for further information.

Data

Policy information about availability of data

All manuscripts must include a data availability statement. This statement should provide the following information, where applicable:

- Accession codes, unique identifiers, or web links for publicly available datasets
- A list of figures that have associated raw data
- A description of any restrictions on data availability

The authors declare that the data supporting the findings of this study are available within the paper and its supplementary information files. If additional data are required, they are available from the corresponding author upon reasonable request.

Field-specific reporting

Please select the one below that is the best fit for your research. If you are not sure, read the appropriate sections before making your selection.

☒ Life sciences ☐ Behavioural & social sciences ☐ Ecological, evolutionary & environmental sciences

Life sciences study design

All studies must disclose on these points even when the disclosure is negative.

Sample size	In flow cytometry, approximately 5000 cells (the number of cells were counted by BD AccuriC6 flow cytometer) were analyzed for each sample. Further increase of cell numbers (e.g. 10000) hardly affects the analysis. In WST-1 assay, we used 4 wells for each transfection condition, which is the usually used sample size in cell line studies.
Data exclusions	For the calculation of the ratio of two fluorescent proteins, we used only cells expressing both fluorescent proteins, because the inclusion of fluorescent-negative cells into analysis increases the effect of auto-fluorescence that hampers to calculate the true ratio of fluorescent proteins. The criteria was pre-established and used in previous studies (e.g. H. Nakanishi et al., Biomaterials, 2017).
Replication	Three to four independent experiments with identical conditions were performed for each figure to check reproducibility. In addition, before these experiments, several preliminary experiments with slightly different conditions were performed, and we obtained similar results.
Randomization	There is no necessity of randomization because identical cell lines were used in each experiment.
Blinding	Blinding was impossible because most of the experiments were performed by one person.

Reporting for specific materials, systems and methods

We require information from authors about some types of materials, experimental systems and methods used in many studies. Here, indicate whether each material, system or method listed is relevant to your study. If you are not sure if a list item applies to your research, read the appropriate section before selecting a response.

Materials & experimental systems

n/a	Involved in the study	
☒	☐	Antibodies
☐	☒	Eukaryotic cell lines
☒	☐	Palaeontology
☒	☐	Animals and other organisms
☒	☐	Human research participants
☒	☐	Clinical data

Methods

n/a	Involved in the study	
☒	☐	ChIP-seq
☐	☒	Flow cytometry
☒	☐	MRI-based neuroimaging

Eukaryotic cell lines

Policy information about cell lines

Cell line source(s)	HeLa cells: American Type Culture Collection (ATCC)<br>201B7-EGFP cells: Dr. Knut Woltjen (CiRA, Kyoto University)
Authentication	HeLa cells were directly obtained from American Type Culture Collection. 201B7-EGFP was directly obtained from Woltjen lab, where the cell line was established.
Mycoplasma contamination	The absence of mycoplasma was confirmed by MycoAlert mycoplasma detection kit (Lonza).
Commonly misidentified lines<br>(See ICLAC register)	No commonly misidentified cell lines were used.

Flow Cytometry

Plots

Confirm that:

☒ The axis labels state the marker and fluorochrome used (e.g. CD4-FITC).

☒ The axis scales are clearly visible. Include numbers along axes only for bottom left plot of group (a 'group' is an analysis of identical markers).

☒ All plots are contour plots with outliers or pseudocolor plots.

☒ A numerical value for number of cells or percentage (with statistics) is provided.

Methodology

Sample preparation	HeLa cells were harvested using Trypsin/EDTA and suspended in DMEM. 201B7-EGFP cells were harvested using 0.5xTrypLe select and suspended in AK02N medium.
Instrument	BD Accuri C6 (BD Biosciences)
Software	For data collection and analysis, CFlow Plus and FlowJo 7.6.5 were used, respectively.
Cell population abundance	As shown in figure 1c, approximately 75-90% of cells were transfected and used for analysis.
Gating strategy	Preliminary FSC/SSC gate were approximately 10^7 and 10^6 in FSC-A and SSC-A, respectively. The gating of fluorescence was determined based on the data of untransfected samples.

☒ Tick this box to confirm that a figure exemplifying the gating strategy is provided in the Supplementary Information.
