# Supplementary material for: Caliciviral protein-based artificial translational activator for mammalian gene circuits with RNA-only delivery
Source: Nat Commun. 2020 Mar 10;11:1297. doi: 10.1038/s41467-020-15061-x (PMC7064597; doi:10.1038/s41467-020-15061-x)
Supplement: Supplementary file 4 — Description of Additional Supplementary Files [file 41467_2020_15061_MOESM4_ESM.pdf]

Title: Supplementary Video 1
Description: Fluorescent time-lapse imaging of cells transfected with MS2CP-1xDmrA and DmrC-VPg(FCV) (A/C heterodimerizer concentration: 500 nM).

Title: Supplementary Video 2
Description: Fluorescent time-lapse imaging of cells transfected with MS2CP-1xDmrA and DmrC-VPg(FCV) (A/C heterodimerizer concentration: 0 nM).
